# Causal association of peripheral immune cell counts with risk of prostate cancer: insights from bidirectional Mendelian randomization

**DOI:** 10.3389/fonc.2024.1374927

**Published:** 2024-11-29

**Authors:** Xiaolu Ren, Li Zhang, Kehua Wang, Fang Li

**Affiliations:** ^1^ Department of Radiology, General Hospital of Ningxia Medical University, Yinchuan, China; ^2^ School of Health Sciences, Universiti Sains Malaysia, Kelantan, Malaysia; ^3^ Department of Urology, People’s Hospital of Wuzhong, Wuzhong, China; ^4^ Department of Vascular Surgery, General Hospital of Ningxia Medical University, Yinchuan, China; ^5^ Department of Neurology, General Hospital of Ningxia Medical University, Yinchuan, China

**Keywords:** causal, peripheral immune cells, prostate cancer risk, insights, bidirectional Mendelian randomization

## Abstract

**Objectives:**

This study aimed to examine the causal relationships between peripheral immune cell counts and prostate cancer, adhering to Mendelian Randomization reporting guidelines for transparency and reproducibility.

**Methods:**

In this study, bidirectional Mendelian randomization (MR) analysis, which includes MR-Egger, weighted median, weighted mode, and inverse variance weighted (IVW) approaches, was utilized to evaluate the bidirectional causal relationship between peripheral immune cell counts and the risk of PCa.

**Results:**

The primary analysis using the IVW method suggests a potential causal association between basophil counts and the risk of prostate cancer (PCa), with an odds ratio (OR) of 1.111 and a 95% confidence interval (CI) of 1.011-1.222 (*P* = 0.028). Conversely, non-causal associations have been observed between other peripheral immune cell types, such as white blood cells, neutrophils, lymphocytes, eosinophils, or monocytes, and the incidence of PCa (*P* values > 0.05). Furthermore, although reverse analysis indicated a causal link between PCa and the counts of leukocytes and neutrophils (OR = 1.013; 95% CI = 1.002–1.225; *P* = 0.018 and OR = 1.013; 95% CI = 1.002–1.025; *P* = 0.019), no causal association was detected between PCa and basophil count (*P* value > 0.050).

**Conclusion:**

This study suggests a potential bidirectional link between peripheral immune cells and prostate cancer, but inconsistencies in Mendelian Randomization methods mean these findings are preliminary and require further investigation.

## Introduction

Prostate cancer (PCa) is more prevalent in elderly males, especially in developed nations. Its occurrence becomes increasingly correlated with age, displaying a notable rise in susceptibility after the age of 50. Key risk factors encompass age, family history, and racial background. The influence of causal factors remains a topic of continuous debate (1), and in terms of genetic patterns, the intricate hereditary nature inherent to PCa distinctly sets it apart from other common types of cancers (2). Genome-wide association studies (GWASs) have pinpointed approximately 150 common risk loci linked to PCa (3, 4), and recent discussions have illuminated various external factors connected to the risk of PCa, such as obesity, metabolic syndrome, and dietary habits (5–7). Given PCa’s substantial global impact, identifying modifiable risk factors becomes of paramount importance to endeavors that curtail its incidence.

Complete blood count serves as a cornerstone in human health assessments, facilitating the straightforward determination of blood cell count and dimensions. Its paramount importance lies in the early detection of the initial indicators of various conditions, achieving the goal of ‘timely identification and prompt intervention (8). Among crucial parameters, white blood cell outcomes hold significant importance. White blood cells comprise a variety of distinct cell types, such as lymphocytes, monocytes, basophils, neutrophils, and eosinophils, which contribute to immune response, defense against invaders, phagocytosis of foreign substances, and antibody synthesis. In particular, the presence of basophils, which belong to a rare subset of circulating granulocytes constituting less than 1% of the total circulating leukocyte counts, is noteworthy. Current investigations propose that basophils potentially exhibit functions that extend beyond their traditionally established roles, and a growing body of evidence indicates that basophils are implicated in a broader spectrum of human diseases spanning allergies, infections, inflammatory conditions, and malignancies (9).

Two-sample Mendelian randomization (MR) is rooted in the fundamental principles of genetic randomization. It employs genetic variations as unbiased proxies and is designed to investigate causal associations between exposure (or risk factors) and outcomes (10, 11). MR explores the causal connections between circulating peripheral blood cells and various conditions, including glaucoma, type 2 diabetes, multiple sclerosis, and reduced narcolepsy (12–15). Concurrently, MR consistently unveils the causal associations of blood lipid, circulating selenium, circulating phosphorus, and circulating free testosterone levels with PCa (16–19). However, a notable gap in MR studies that examine the causal relationship between circulating immune cell count and the risk of PCa remains.

In the current investigation, we conducted a two-sample MR analysis utilizing the GWAS database to assess the genetic causality between peripheral immune cell counts and the risk of PCa.

## Materials and methods

### Study design

**Figure 1:**
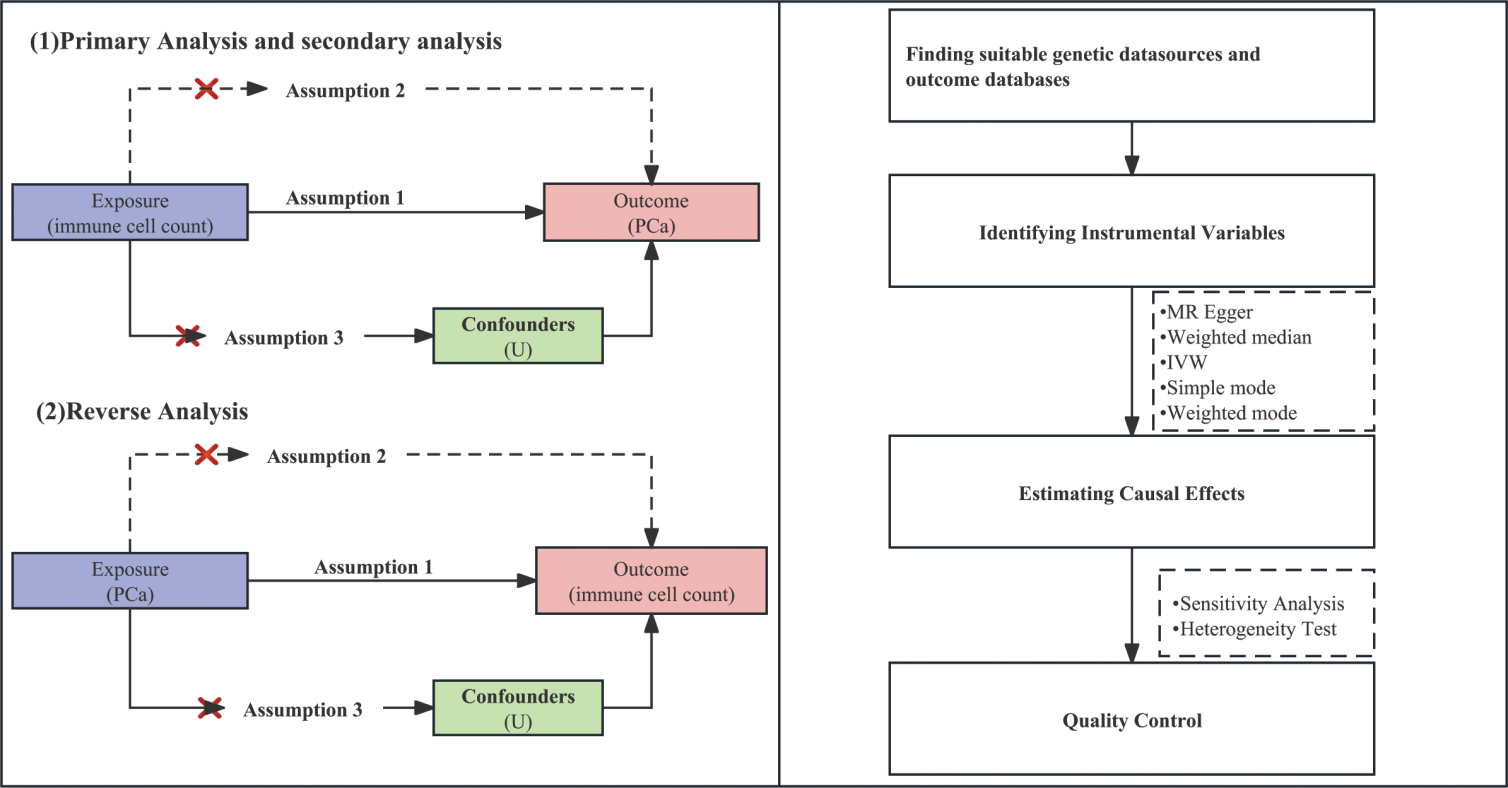
Design and main assumptions of our Mendelian randomization study.

The groundbreaking research conducted by Davey Smith and Hemani (2014) established the fundamental basis for the implementation of two-sample MR, which is a framework that enables us to leverage the advantages of publicly available genetic summary data (10). At the same time, our study strictly adhered to “Strengthening the Reporting of Observational Studies in Epidemiology-Mendelian Randomization (STROBE-MR)” guidelines published in the BMJ, which were formulated according to the framework established by the Enhancing the QUAlity and Transparency Of health Research (EQUATOR) network (20). By integrating the STROBE-MR guidelines into our methodology, we aim to enhance the clarity and assessment of our research. By strategically employing genetic variations as instrumental variables (IVs) to quantify immune cell counts, our primary objective is centered on mitigating the potential challenges of reverse causation and confounding biases that frequently affect traditional observational investigations. Our MR analysis relies on three essential assumptions: ① IV assumption: We postulate that the selected IVs inherently exhibit connections with peripheral immune cell counts. ② Independence assumption: The IVs maintain their independence from conceivable confounding variables that might obscure the causal relationship between immune cell traits and the onset of PCa. ③ Exclusion restriction assumption: The IVs solely impact PCa outcomes by influencing the attributes of immune cells present in peripheral blood. The comprehensive framework of this study’s design is visually presented in **Figure 1**.

### Data sources

Effect estimates associated with single-nucleotide polymorphisms (SNPs) influencing peripheral blood cells encompassing various types, such as white blood cells, lymphocytes, monocytes, neutrophils, eosinophils, and basophils, were derived from an extensive meta-analysis using blood cell consortium. This all-encompassing study incorporated data from a substantial cohort of 563,946 individuals with European lineage (21). Simultaneously, summary metrics regarding GWAS linked to PCa involving individuals of European descent were sourced from the IEU Open GWAS repository. This particular GWAS comprises 140,254 participants (consisting of 79,148 cases and 61,106 controls) and serves as the primary dataset for the study’s outcomes (3). Notably, this investigation exclusively relied upon publicly accessible GWAS summary data, eliminating the necessity of seeking ethical approval or utilizing individual-level data. Each trait that we selected is shown in **Supplementary Tables S1**–**7**.

### Selection of genetic instrumental variables

We adhered to a stringent set of criteria to ensure the reliability and independence of the selected SNPs as potential IVs. These SNPs must exceed statistically significant genome-wide association thresholds that are specifically related to relevant peripheral cell count traits. This study employed three primary methodologies: primary analyses, secondary analyses, and reverse analysis. Primary analysis and reverse analysis employ a rigorous *P* value threshold of <5 × 10^−8^, whereas secondary analysis uses a more stringent threshold (*P* < 1 × 10^−12^). To further enhance the independence of the variables, we applied linkage disequilibrium criteria (*r* ^2^ = 0.001; 10 Mb). Then, we established a threshold to exclude SNPs with effect allele frequencies exceeding 0.420. This criterion ensures that the selected SNPs can represent a majority of the population. To ensure the robustness of the IV selection process, we excluded SNPs with palindromic sequences and ambiguous intermediate effect frequencies. To assess the strength of the IVs, we excluded SNPs with an F-statistic less than 10.

### Statistical analysis

A schematic summary of the statistical analysis is provided in **Figure 1**. Our approach revolves around the utilization of two-sample MR methodologies, which encompass a variety of techniques, including MR egger, weighted median and inverse variance weighted (IVW), simple mode, and weighted mode. We assessed the association between exposure and outcome by means of odds ratios (ORs), standardizing them by computing standard deviation increments for each exposure factor. Our investigation employed various analytical strategies and quality control measures to ensure the robustness and validity of our findings, and we conducted sensitivity, heterogeneity, changed threshold, and bidirectional analyses to evaluate the causal link between immune cell count and the risk of PCa. The robustness of causal estimates to potential sources of bias was assessed through sensitivity analysis, and the robustness of MR results was assessed through leave-one-out analysis, in which one SNP was systematically excluded at a time. Heterogeneity analysis helped us understand whether the causal effect varies across different subpopulations. We employed IVW methods along with Cochran’s Q statistics to assess heterogeneity and MR-Egger intercept to evaluate pleiotropy among individual SNPs. To visually inspect and evaluate the results, funnel plots and scatter plots were used as tools to identify potential bias and publication bias. Additionally, we introduced a third research method: reverse analysis. This approach aids in addressing reverse causality and investigating whether or not PCa itself has an impact on peripheral immune cell counts. Our analyses were conducted using the Two Sample MR package (version 0.5.6), proficiently implemented within the R programming environment (version 4.3.0).

## Results

### Effect of peripheral immune cell counts on the risk of PCa (primary analysis and secondary analysis)

**Figure 2:**
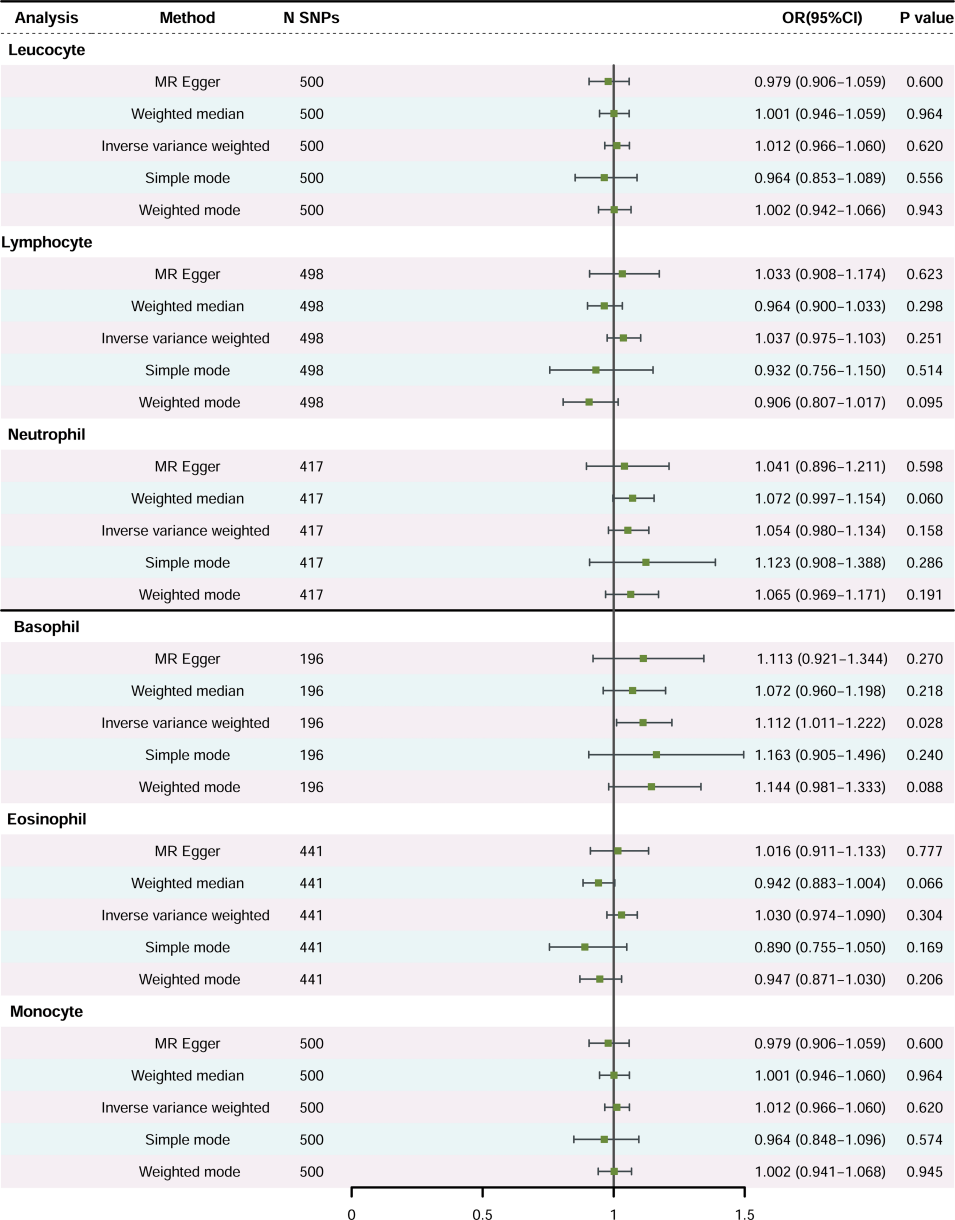
The causal effects of peripheral immune cell counts on risk of prostate cancer using two-sample Mendelian randomization methods (Primary Analysis). SNP, single-nucleotide polymorphisms; OR, odds ratio; CI, confidence interval.

Primary Analysis: **Figure 2** illustrates the association between peripheral immune cell counts and the risk of PCa, utilizing the IVW method. A significant association was observed, indicating that elevated basophil cell counts are correlated with an increased susceptibility to PCa, with an OR of 1.111, 95% confidence interval (CI) from 1.012 to 1.222, and a *P*-value of 0.028. Conversely, there was no significant correlation between PCa and counts of white blood cells, neutrophils, lymphocytes, eosinophils, or monocytes (*P*-values > 0.05).

Secondary Analysis: This analysis aimed to validate and potentially strengthen the correlations observed in the primary analysis. However, the findings did not reveal stronger associations (**Table 1**). The OR between basophil count and PCa risk was 1.074, with a 95% confidence interval ranging from 1.012 to 1.178, and the correlation did not reach statistical significance (*P*-value of 0.162).

**Table 1:** Mendelian randomization estimates of the association between peripheral immune cell counts and risk of prostate cancer (Secondary Analysis).

Analysis	Method	nSNP	*P* value	OR(95%CI)
leucocyte				
	MR Egger	297.000	0.568	1.045 (0899-1.214)
	Weighted median	297.000	0.195	1.048 (0.975-1.127)
	Inverse variance weighted	297.000	0.507	1.023 (0.957-1.093)
	Simple mode	297.000	0.457	1.085 (0.875-1.346)
	Weighted mode	297.000	0.579	1.037 (0.913-1.178)
lymphocyte				
	MR Egger	286.000	0.897	1.001 (0.873-1.168)
	Weighted median	286.000	0.125	0.943 (0.875-1.016)
	Inverse variance weighted	286.000	0.754	1.010 (0.949-1.075)
	Simple mode	286.000	0.313	0.902 (0.738-1.102)
	Weighted mode	286.000	0.068	0.890 (0.787-1.008)
neutrophil				
	MR Egger	235.000	0.233	1.126 (0.927-1.367)
	Weighted median	235.000	0.139	1.060 (0.981-1.146)
	Inverse variance weighted	235.000	0.496	1.031 (0.944-1.126)
	Simple mode	235.000	0.374	1.096(0.896-1.341)
	Weighted mode	235.000	0.284	1.058 (0.954-1.173)
basophil				
	MR Egger	104.000	0.159	1.165 (0.944-1.439)
	Weighted median	104.000	0.255	1.071 (0.952-1.204)
	Inverse variance weighted	104.000	0.162	1.074 (1.012-1.178)
	Simple mode	104.000	0.237	1.162 (0.972-1.187)
	Weighted mode	104.000	0.117	1.142 (0.969-1.347)
eosinophil				
	MR Egger	261.000	0.663	1.028 (0.908-1.164)
	Weighted median	261.000	0.077	0.942 (0.881-1.006)
	Inverse variance weighted	261.000	0.643	1.014 (0.956-1.075)
	Simple mode	261.000	0.305	0.919 (0.783-1.079)
	Weighted mode	261.000	0.149	0.937 (0.857-1.023)
monocyte				
	MR Egger	312.000	0.754	0.986 (0.905-1.075)
	Weighted median	312.000	0.965	1.001 (0.944-1.062)
	Inverse variance weighted	312.000	0.857	0.996 (0.948-1.045)
	Simple mode	312.000	0.794	0.982 (0.857-1.126)
	Weighted mode	312.000	0.638	1.017 (0.948-1.092)

SNP, single-nucleotide polymorphisms; OR, odds ratio; CI, confidence interval.

### Evaluation of heterogeneity and pleiotropy (primary analysis and secondary analysis)

A detailed summary of our findings, including heterogeneity and pleiotropy assessments, is presented in **Table 2** and **Supplementary Table S8**. Cochran’s Q test revealed high heterogeneity among the six analyzed peripheral immune cell types (P values near zero), while the MR-Egger intercept test showed no significant pleiotropic effects (*P* values > 0.05), affirming the validity of the genetic instruments used for causal estimation in our MR analysis. Visual assessments of the funnel plot and scatter diagram (**Supplementary Figures S1**–**12**) found no significant directional pleiotropy, bolstering the reliability and accuracy of our MR study’s findings.

**Table 2:** Heterogeneity test and horizontal pleiotropy test of the association between peripheral immune cell counts and risk of prostate cancer (Primary analysis).

Analysis	MR Heterogeneity Test				MR Horizontal Pleiotropy Test		
	Method	Q	Q-df	Q-pval	Egger-intercept	se	*P* value
**leucocyte**	MR Egger	1167.504	480.000	<0.001	-1.20E-04	0.001	0.934
	Inverse Variance Weighted	1167.521	481.000	<0.001			
**lymphocyte**	MR Egger	1447.754	496.000	<0.001	1.12E-04	0.002	0.943
	Inverse Variance Weighted	1447.769	497.000	<0.001			
**neutrophil**	MR Egger	1363.758	415.000	<0.001	3.17E-04	0.002	0.858
	Inverse Variance Weighted	1363.863	416.000	<0.001			
**basophil**	MR Egger	494.941	194.000	<0.001	-2.37E-05	0.002	0.992
	Inverse Variance Weighted	494.942	195.000	<0.001			
**eosinophil**	MR Egger	1146.511	439.000	<0.001	4.41E-04	0.002	0.774
	Inverse Variance Weighted	1146.728	440.000	<0.001			
**monocyte**	MR Egger	1200.048	498.000	<0.001	1.20E-03	0.001	0.306
	Inverse Variance Weighted	1202.581	499.000	<0.001			

Q, Quantile; df, degrees of freedom; se, standard error.

### Effect of the risk of PCa on peripheral immune cell counts (reverse analysis)

In our reverse analysis, presented in **Table 3**, we investigated the potential influence of PCa risk on peripheral immune cell counts. The analysis revealed a statistically significant association indicating that heightened susceptibility to PCa correlates with increased counts of leucocytes (IVW method: OR = 1.013; 95% CI 1.002 to 1.225; *P* = 0.018) and neutrophils (IVW method: OR = 1.013; 95% CI 1.002 to 1.025; *P* = 0.019). Conversely, our findings did not demonstrate a significant relationship between PCa risk and basophil counts (IVW method: OR = 1.008; 95% CI 0.997–1.019; *P* = 0.142).

**Table 3:** MR Estimates of PCa Risk Effects on Peripheral Immune Cell Counts (Reverse analysis).

Analysis	Method	nSNPs	OR(95%CI)	*P* value
**leucocyte**	MR Egger	120	1.016 (0.994-1.038)	0.151
	Weighted median	120	1.012 (1.004-1.020)	0.002
	Inverse variance weighted	120	1.013 (1.002-1.025)	0.018
	Simple mode	120	1.000 (0.986-1.015)	0.974
	Weighted mode	120	1.008 (0.999-1.017)	0.071
**lymphocyte**	MR Egger	120	1.005 (0.982-1.027)	0.690
	Weighted median	120	1.000 (0.992-1.008)	0.962
	Inverse variance weighted	120	1.003 (0.991-1.014)	0.644
	Simple mode	120	1.003 (0.990-1.016)	0.702
	Weighted mode	120	0.999 (0.991-1.007)	0.807
**neutrophil**	MR Egger	120	1.015 (0.993-1.037)	0.178
	Weighted median	120	1.005 (0.997-1.012)	0.209
	Inverse variance weighted	120	1.013 (1.002-1.025)	0.019
	Simple mode	120	0.999 (0.983-1.016)	0.926
	Weighted mode	120	1.006(0.998-1.014)	0.152
**basophil**	MR Egger	120	1.006 (0.985-1.027)	0.598
	Weighted median	120	1.001 (0.993-1.009)	0.840
	Inverse variance weighted	120	1.008 (0.997-1.019)	0.142
	Simple mode	120	0.997 (0.981-1.013)	0.694
	Weighted mode	120	1.003 (0.993-1.014)	0.516
**eosinophil**	MR Egger	120	1.002 (0.972-1.034)	0.887
	Weighted median	120	1.005 (0.997-1.014)	0.238
	Inverse variance weighted	120	1.008(0.991-1.024)	0.365
	Simple mode	120	1.000 (0.981-1.020)	0.967
	Weighted mode	120	1.005 (0.997-1.014)	0.210
**monocyte**	MR Egger	120	1.029 (1.002-1.057)	0.034
	Weighted median	120	1.007(0.999-1.015)	0.087
	Inverse variance weighted	120	1.013 (1.000-1.026)	0.054
	Simple mode	120	0.989 (0.970-1.007)	0.229
	Weighted mode	120	1.008 (1.000-1.016)	0.040

SNP, single-nucleotide polymorphisms; OR, Odds Ratio; CI, confidence interval.

## Discussion

Our preliminary analysis suggests a possible association between peripheral immune cell counts and the risk of developing PCa, with an observed increase in basophil counts. The *P*-values from Cochran’s Q test approached zero, indicating substantial heterogeneity among the studied immune cell types. Furthermore, results from the MR-Egger intercept test, showing P-values greater than 0.05, found no significant horizontal pleiotropy. In an expanded reverse analysis, no causal relationship was detected between PCa exposure and variations in basophil cell counts. However, a strong correlation was observed between PCa exposure and increased counts of leukocytes and neutrophils.

This preliminary analysis discovery aligns with the research conducted by Hadadi et al., who noted that an increase in eosinophil counts is significantly negatively correlated with both overall survival and progression-free survival in patients with metastatic hormone-sensitive prostate cancer, suggesting that this cell type might have a detrimental impact on clinical outcomes (22). However, the studies by Hayashi et al. were unable to establish a definitive link between eosinophil levels and the Gleason score (23). This discrepancy may indicate the varied biological roles of eosinophils in different stages or subtypes of prostate cancer.

Recent studies elucidate the intricate roles of cytokines and chemokines secreted by basophils in modulating the tumor microenvironment. When basophils are co-cultured with fibroblasts, they secrete interleukin-4 (IL-4) and tumor necrosis factor-alpha (TNF-α), which subsequently trigger the expression of CCL11 in the fibroblasts (24), affecting their migration and proliferation dynamics. In environments characterized by hormone-resistant prostate cancer (25), research indicates that increased IL-4 levels substantially boost the clonogenic capacity of cancer stem cell-like entities (26) and augment the proliferation of androgen-sensitive LNCaP cells via IL-4 overexpression (27). Additionally, TNF-α is identified as a pivotal contributor to prostate cancer pathogenesis (28), closely associated with detrimental pathological characteristics of PCa (29). Moreover, the serum protein biomarker CCL11 (eotaxin-1), a potent chemotactic agent for basophils, is elevated in individuals with prostate cancer (30), highlighting its role in regulating immune responses and advancing tumor progression. Collectively, these findings underscore the critical influence of basophils in the development and progression of prostate cancer, demonstrating that their role surpasses traditional immunoregulatory functions.

In the expanded reverse analysis, no causal link was detected between PCa exposure and variations in basophil cell counts. This result implies that prostate cancer may not directly influence basophil cell count alterations. However, we found a strong correlation between PCa as the exposure factor and the resulting increase in leukocyte and neutrophil cell counts. Previous controlled studies have confirmed the increase in leukocyte (31) and neutrophil (32) counts associated with PCa. Correspondingly, analysis of 966 males suspected of harboring prostate cancer revealed a correlation between changes in white blood cell counts and high Gleason scores (23). These observations highlight the significance of systemic inflammatory responses in the advancement of prostate cancer. These findings not only corroborate our results but also deepen our understanding of the interplay between inflammation and prostate cancer progression.

The primary strength of this study lies in its innovation. This is the first time that the MR approach has been employed to explore the potential causal relationships between peripheral immune cell counts and PCa risk using extensive GWAS data. Unlike earlier studies, such as those by Hadidi et al. (22), which primarily analyzed the correlation of basophils through clinical data, this research utilizes genetic tools and a broad spectrum of genetic variation data, offering a completely new perspective on the interactions between peripheral immune cell counts and PCa risk.

However, we must acknowledge the possibility of unmeasured or residual confounding factors in our primary analysis. The significant heterogeneity observed among different immune cell types underscores the complexity of immune responses in the context of PCa. While efforts have been made to mitigate the impact of horizontal pleiotropy, the complexity and unpredictability of genetic variant biology mean that completely eliminating this influence remains a significant challenge. Therefore, achieving greater statistical robustness requires larger sample sizes and more advanced methodologies. Furthermore, it is important to recognize that the immune system is a dynamic and intricate network of cells and molecules. The relationship between immune cell counts and the risk of prostate cancer may be influenced by a variety of factors, including genetic predispositions, environmental exposures, and lifestyle choices. Although our study sheds light on potential causal associations, it represents only one piece of the complex puzzle.

A more stringent secondary analysis, which set a higher *P*-value threshold (*P* < 1 × 10^-12^), failed to confirm this association, possibly due to insufficient statistical power, sample heterogeneity, randomness from multiple testing, inadequate efficacy of genetic tools, and data quality issues. These discrepancies necessitate a cautious interpretation of the results and may require further research to validate these findings. The challenges and differences encountered in our study highlight important considerations that future research designs in this field must address. It is recommended that future studies increase sample sizes, improve instrumentation, enhance validation, explore mechanisms, utilize longitudinal data, conduct stratified evaluations, and employ meta-analysis to comprehensively understand the relationship between peripheral immune cell counts and prostate cancer.

## Conclusion

In summary, our MR analysis provides genetic evidence for a potential link between peripheral immune cell counts and the risk of PCa. However, given the inconsistencies in results across different *P*-value thresholds in MR methods, we should interpret these findings with caution. Future studies should include large-scale randomized controlled trials to obtain more conclusive evidence and gain a deeper understanding of this relationship.

## Data Availability

The original contributions are included in the article/**Supplementary Material**, with access links in **Supplementary Table S7**. For inquiries, contact the corresponding author.

## References

[1] Zi H He SH Leng XY Xu XF Huang Q Weng H. Global, regional, and national burden of kidney, bladder, and prostate cancers and their attributable risk factors, 1990-2019. Mil Med Res. (2021) 8:60. doi: 10.1186/s40779-021-00354-z 34819142 PMC8611255

[2] Dupont WD Breyer JP Plummer WD Chang SS Cookson MS Smith JA. 8q24 genetic variation and comprehensive haplotypes altering familial risk of prostate cancer. Nat Commun. (2020) 11:1523. doi: 10.1038/s41467-020-15122-1 32251286 PMC7089954

[3] Schumacher FR Al Olama AA Berndt SI Benlloch S Ahmed M Saunders EJ. Association analyses of more than 140,000 men identify 63 new prostate cancer susceptibility loci. Nat Genet. (2018) 50:928–36. doi: 10.1038/s41588-018-0142-8 PMC6568012 29892016

[4] Spisak S Tisza V Nuzzo PV Seo JH Pataki B Ribli D. A biallelic multiple nucleotide length polymorphism explains functional causality at 5p15.33 prostate cancer risk locus. Nat Commun. (2023) 14:5118. doi: 10.1038/s41467-023-40616-z 37612286 PMC10447552

[5] Kazmi N Haycock P Tsilidis K Lynch BM Truong T. Appraising causal relationships of dietary, nutritional and physical-activity exposures with overall and aggressive prostate cancer: two-sample Mendelian-randomization study based on 79 148 prostate-cancer cases and 61 106 controls. Int J Epidemiol. (2020) 49:587–96. doi: 10.1093/ije/dyz235 31802111

[6] Lifshitz K Ber Y Margel D. Role of metabolic syndrome in prostate cancer development. Eur Urol Focus. (2021) 7:508–12. doi: 10.1016/j.euf.2021.04.022 33994167

[7] Perez-Cornago A Smith-Byrne K Hazelwood E Watling CZ Martin S Frayling T. Genetic predisposition to metabolically unfavourable adiposity and prostate cancer risk: A Mendelian randomization analysis. Cancer Med. (2023) 12:16482–9. doi: 10.1002/cam4.6220 PMC10469819 37305903

[8] Tefferi A Hanson CA Inwards DJ. How to interpret and pursue an abnormal complete blood cell count in adults. Mayo Clin Proc. (2005) 80:923–36. doi: 10.4065/80.7.923 PMC7127472 16007898

[9] Chen K Hao Y Guzmán M Li G Cerutti A. Antibody-mediated regulation of basophils: emerging views and clinical implications. Trends Immunol. (2023) 44:408–23. doi: 10.1016/j.it.2023.04.003 PMC10219851 37147229

[10] Davey Smith G Hemani G. Mendelian randomization: genetic anchors for causal inference in epidemiological studies. Hum Mol Genet. (2014) 23:R89–98. doi: 10.1093/hmg/ddu328 PMC4170722 25064373

[11] Emdin CA Khera AV Kathiresan S. Mendelian randomization. JAMA. (2017) 318:1925–6. doi: 10.1001/jama.2017.17219 29164242

[12] He D Liu L Shen D Zou P Cui L. The effect of peripheral immune cell counts on the risk of multiple sclerosis: A mendelian randomization study. Front Immunol. (2022) 13:867693. doi: 10.3389/fimmu.2022.867693 35619713 PMC9128528

[13] Li A Li H Xie J Xie J Liao W Song L. Higher basophil count decreases narcolepsy risk: a Mendelian randomization study. Neurol Sci. (2022) 43:5575–80. doi: 10.1007/s10072-022-06123-7 35554757

[14] Li J Niu Q Wu A Zhang Y Hong L Wang H. Causal relationship between circulating immune cells and the risk of type 2 diabetes: a Mendelian randomization study. Front Endocrinol (Lausanne). (2023) 14:1210415. doi: 10.3389/fendo.2023.1210415 37305035 PMC10247959

[15] Song DJ Fan B Li GY. Blood cell traits and risk of glaucoma: A two-sample mendelian randomization study. Front Genet. (2023) 14:2023.1142773. doi: 10.3389/fgene.2023.1142773 PMC10130872 37124610

[16] Bull CJ Bonilla C Holly JM Perks CM Davies N Haycock P. Blood lipids and prostate cancer: a Mendelian randomization analysis. Cancer Med. (2016) 5:1125–36. doi: 10.1002/cam4.695 PMC4924371 26992435

[17] Lv L Ye D Chen J Qian Y Fu AN Song J. Circulating phosphorus concentration and risk of prostate cancer: a Mendelian randomization study. Am J Clin Nutr. (2022) 115:534–43. doi: 10.1093/ajcn/nqab342 34617559

[18] Watts EL Perez-Cornago A Fensom GK Smith-Byrne K Noor U Andrews CD. Circulating free testosterone and risk of aggressive prostate cancer: Prospective and Mendelian randomisation analyses in international consortia. Int J Cancer. (2022) 151:1033–46. doi: 10.1002/ijc.34116 PMC7613289 35579976

[19] Yarmolinsky J Bonilla C Haycock PC Langdon RJQ Lotta LA Langenberg C. Circulating selenium and prostate cancer risk: A mendelian randomization analysis. JNCI: J Natl Cancer Institute. (2018) 110:1035–8. doi: 10.1093/jnci/djy081 PMC6136927 29788239

[20] Skrivankova VW Richmond RC Woolf BAR Davies NM Swanson SA VanderWeele TJ. Strengthening the reporting of observational studies in epidemiology using mendelian randomisation (STROBE-MR): explanation and elaboration. BMJ (Clinical Res ed.). (2021) 375:n2233. doi: 10.1136/bmj.n2233 PMC8546498 34702754

[21] Vuckovic D Bao EL Akbari P Lareau CA Mousas A Jiang T. The polygenic and monogenic basis of blood traits and diseases. Cell. (2020) 182:1214–1231.e1211. doi: 10.1016/j.cell.2020.08.008 32888494 PMC7482360

[22] Hadadi A Smith KE Wan L Brown JR Russler G Yantorni L. Baseline basophil and basophil-to-lymphocyte status is associated with clinical outcomes in metastatic hormone sensitive prostate cancer. Urologic Oncol. (2022) 40:271.e9–271.e18. doi: 10.1016/j.urolonc.2022.03.016 PMC9117505 35466038

[23] Hayashi T Fujita K Tanigawa G Kawashima A Nagahara A Ujike T. Serum monocyte fraction of white blood cells is increased in patients with high Gleason score prostate cancer. Oncotarget. (2017) 8:35255–61. doi: 10.18632/oncotarget.13052 PMC5471051 27823973

[24] Nakashima C Otsuka A Kabashima K. Recent advancement in the mechanism of basophil activation. J Dermatol Sci. (2018) 91:3–8. doi: 10.1016/j.jdermsci.2018.03.007 29602578

[25] Wise GJ Marella VK Talluri G Shirazian D. Cytokine variations in patients with hormone treated prostate cancer. J Urol. (2000) 164:722–5. doi: 10.1097/00005392-200009010-00024 10953133

[26] Nappo G Handle F Santer FR McNeill RV Seed RI Collins AT. The immunosuppressive cytokine interleukin-4 increases the clonogenic potential of prostate stem-like cells by activation of STAT6 signalling. (2017) Oncogenesis. 6:e342. doi: 10.1038/oncsis.2017.23 28553931 PMC5523058

[27] Lee SO Lou W Hou M Onate SA Gao AC. Interleukin-4 enhances prostate-specific antigen expression by activation of the androgen receptor and Akt pathway. Oncogene. (2003) 22:7981–8. doi: 10.1038/sj.onc.1206735 12970746

[28] Michalaki V Syrigos K Charles P Waxman J. Serum levels of IL-6 and TNF-alpha correlate with clinicopathological features and patient survival in patients with prostate cancer. Br J Cancer. (2004) 90:2312–6. doi: 10.1038/sj.bjc.6601814 PMC2409519 15150588

[29] Chadha KC Miller A Nair BB Schwartz SA Trump DL Underwood W. New serum biomarkers for prostate cancer diagnosis. Clin Cancer Investig J. (2014) 3:72–9. doi: 10.4103/2278-0513.125802 PMC4292911 25593898

[30] Agarwal M He C Siddiqui J Wei JT Macoska JA. CCL11 (eotaxin-1): a new diagnostic serum marker for prostate cancer. Prostate. (2013) 73:573–81. doi: 10.1002/pros.22597 PMC3594486 23059958

[31] Bahig H Taussky D Delouya G Nadiri A Gagnon-Jacques A Bodson-Clermont P. Neutrophil count is associated with survival in localized prostate cancer. BMC Cancer. (2015) 15:594. doi: 10.1186/s12885-015-1599-9 26292807 PMC4546219

[32] Maynard JP Godwin TN Lu J Vidal I Lotan TL De Marzo AM. Localization of macrophage subtypes and neutrophils in the prostate tumor microenvironment and their association with prostate cancer racial disparities. Prostate. (2022) 82:1505–19. doi: 10.1002/pros.24424 35971807

