# Supplementary material for: Causal association of peripheral immune cell counts with risk of prostate cancer: insights from bidirectional Mendelian randomization
Source: Front Oncol. 2024 Nov 29;14:1374927. doi: 10.3389/fonc.2024.1374927 (PMC11638012; doi:10.3389/fonc.2024.1374927)

Causal Association of Peripheral Immune Cell Counts with Risk of Prostate Cancer: Insights from Bidirectional Mendelian Randomization

**Supplementary Figure 1.** Scatter plot (A), forest plot (B), and “leave-one-out” analysis (C) for MR analysis of Leucocyte Counts and PCa risk, funnel plot (D) (Primary analysis).

**Supplementary Figure 2.** Scatter plot (A), forest plot (B), and “leave-one-out” analysis (C) for MR analysis of Lymphocyte Counts and PCa risk, funnel plot (D) (Primary analysis).

**Supplementary Figure 3.** Scatter plot (A), forest plot (B), and “leave-one-out” analysis (C) for MR analysis of Neutrophil Counts and PCa risk, funnel plot (D) (Primary analysis).

**Supplementary Figure 4.** Scatter plot (A), forest plot (B), and “leave-one-out” analysis (C) for MR analysis of Basophil Counts and PCa risk, funnel plot (D) (Primary analysis).

**Supplementary Figure 5.** Scatter plot (A), forest plot (B), and “leave-one-out” analysis (C) for MR analysis of Eosinophil Counts and PCa risk, funnel plot (D) (Primary analysis).

**Supplementary Figure 6.** Scatter plot (A), forest plot (B), and “leave-one-out” analysis (C) for MR analysis of Monocyte Counts and PCa risk, funnel plot (D) (Primary analysis).

**Supplementary Figure 7.** Scatter plot (A), forest plot (B), and “leave-one-out” analysis (C) for MR analysis of Leucocyte Counts and PCa risk, funnel plot (D) (Secondary analysis).

**Supplementary Figure 8.** Scatter plot (A), forest plot (B), and “leave-one-out” analysis (C) for MR analysis of Lymphocyte Counts and PCa risk, funnel plot (D) (Secondary analysis).

**Supplementary Figure 9.** Scatter plot (A), forest plot (B), and “leave-one-out” analysis (C) for MR analysis of Neutrophil Counts and PCa risk, funnel plot (D) (Secondary analysis).

**Supplementary Figure 10.** Scatter plot (A), forest plot (B), and “leave-one-out” analysis (C) for MR analysis of Basophil Counts and PCa risk, funnel plot (D) (Secondary analysis).

**Supplementary Figure 11.** Scatter plot (A), forest plot (B), and “leave-one-out” analysis (C) for MR analysis of Eosinophil Counts and PCa risk, funnel plot (D) (Secondary analysis).

**Supplementary Figure 12.** Scatter plot (A), forest plot (B), and “leave-one-out” analysis (C) for MR analysis of Monocyte Counts and PCa risk, funnel plot (D) (Secondary analysis).

**Supplementary Figure 13.** Scatter plot (A), forest plot (B), and “leave-one-out” analysis (C) for MR analysis of PCa risk on Leucocyte Counts funnel plot (D) (Reverse analysis).

**Supplementary Figure 14.** Scatter plot (A), forest plot (B), and “leave-one-out” analysis (C) for MR analysis of PCa risk on Lymphocyte Counts, funnel plot (D) (Reverse analysis).

**Supplementary Figure 15.** Scatter plot (A), forest plot (B), and “leave-one-out” analysis (C) for MR analysis of PCa risk on Neutrophil Counts, funnel plot (D) (Reverse analysis).

**Supplementary Figure 16.** Scatter plot (A), forest plot (B), and “leave-one-out” analysis (C) for MR analysis of PCa risk on Basophil Counts, funnel plot (D) (Reverse analysis).

**Supplementary Figure 17.** Scatter plot (A), forest plot (B), and “leave-one-out” analysis (C) for MR analysis of PCa risk on Eosinophil Count, funnel plot (D) (Reverse analysis).

**Supplementary Figure 18.** Scatter plot (A), forest plot (B), and “leave-one-out” analysis (C) for MR analysis of PCa risk on Monocyte Counts, funnel plot (D) (Reverse analysis).

**Supplementary Figure 1.** Scatter plot (A), forest plot (B), and “leave-one-out” analysis (C) for MR analysis of Leucocyte Counts and PCa risk, funnel plot (D) (Primary analysis).


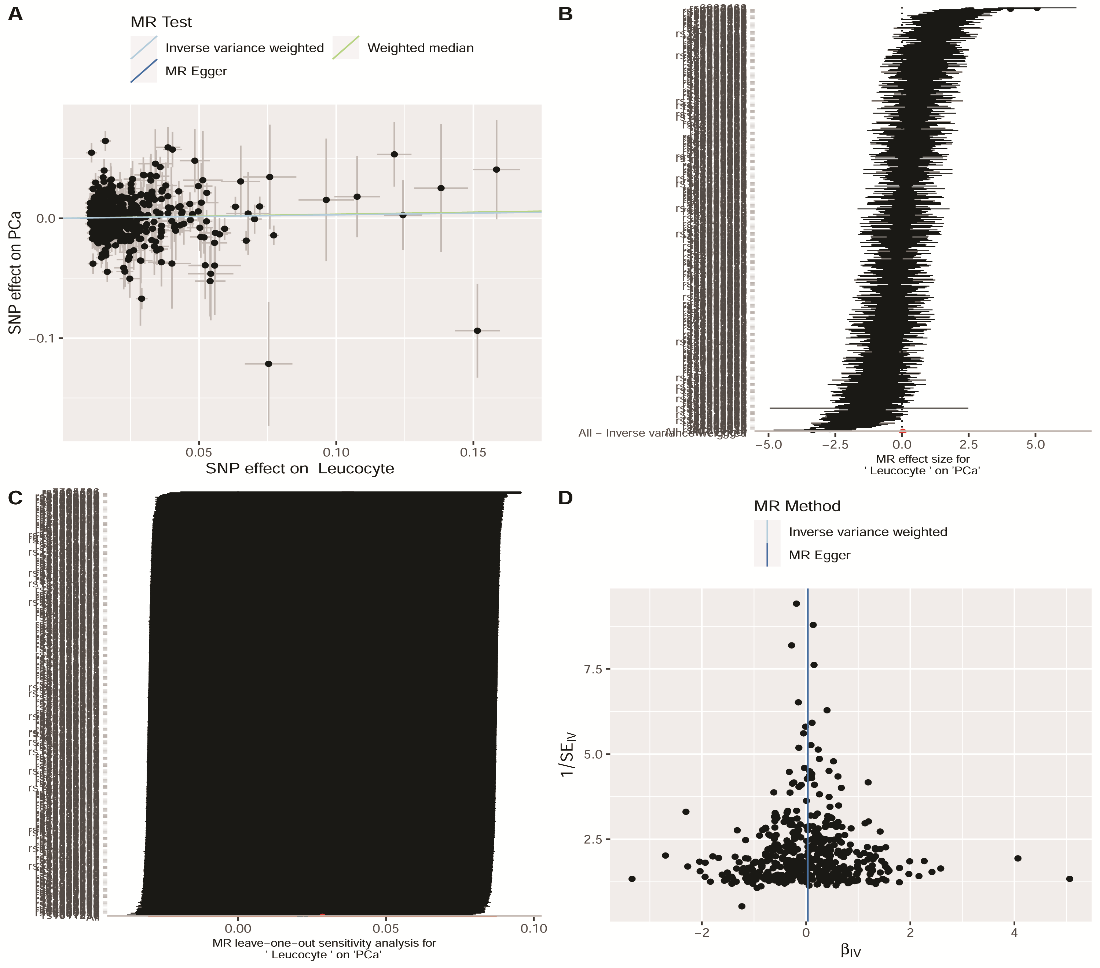


**Supplementary Figure 2.** Scatter plot (A), forest plot (B), and “leave-one-out” analysis (C) for MR analysis of Lymphocyte Counts and PCa risk, funnel plot (D) (Primary analysis).


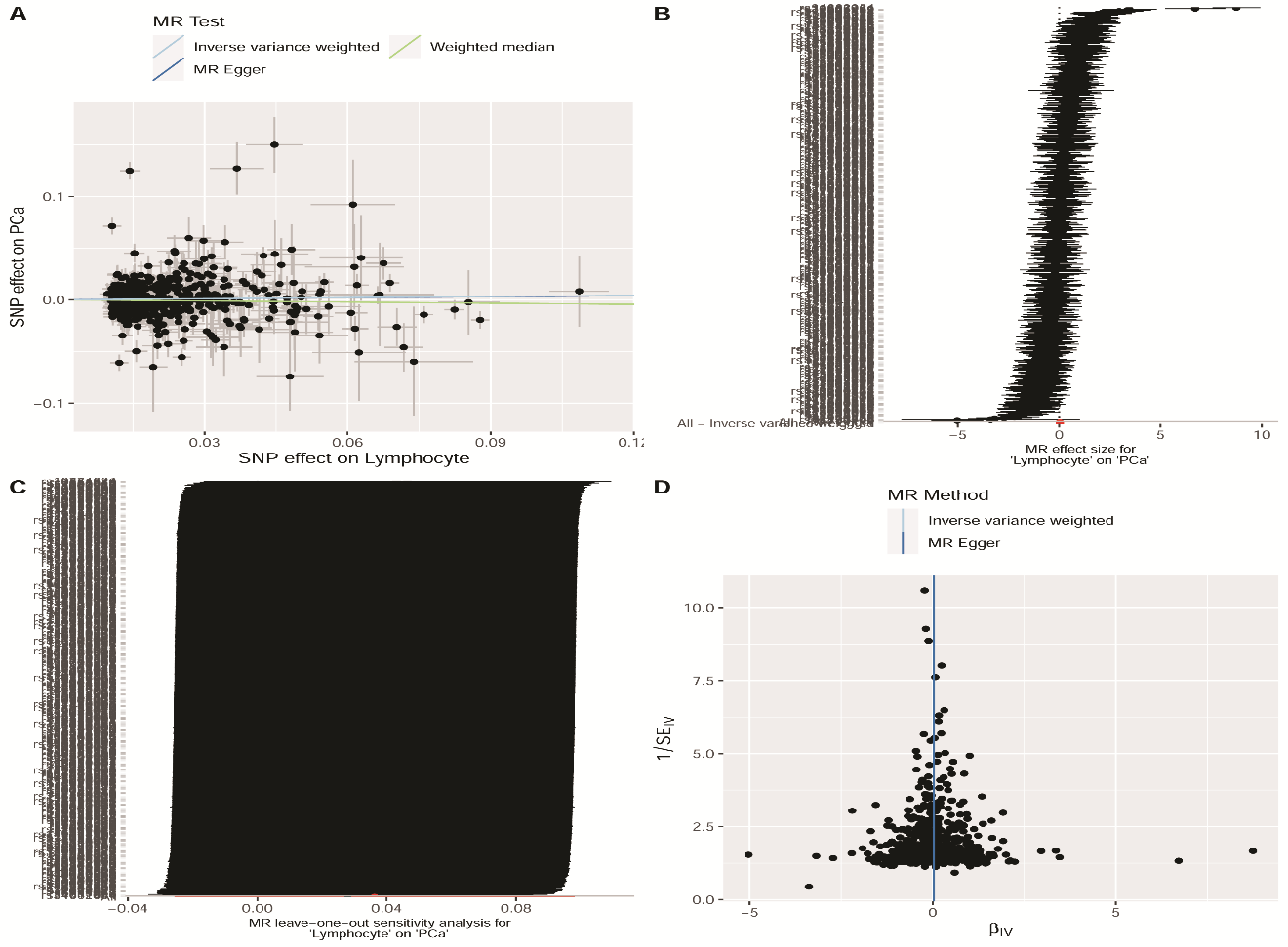


**Supplementary Figure 3.** Scatter plot (A), forest plot (B), and “leave-one-out” analysis (C) for MR analysis of Neutrophil Counts and PCa risk, funnel plot (D) (Primary analysis).


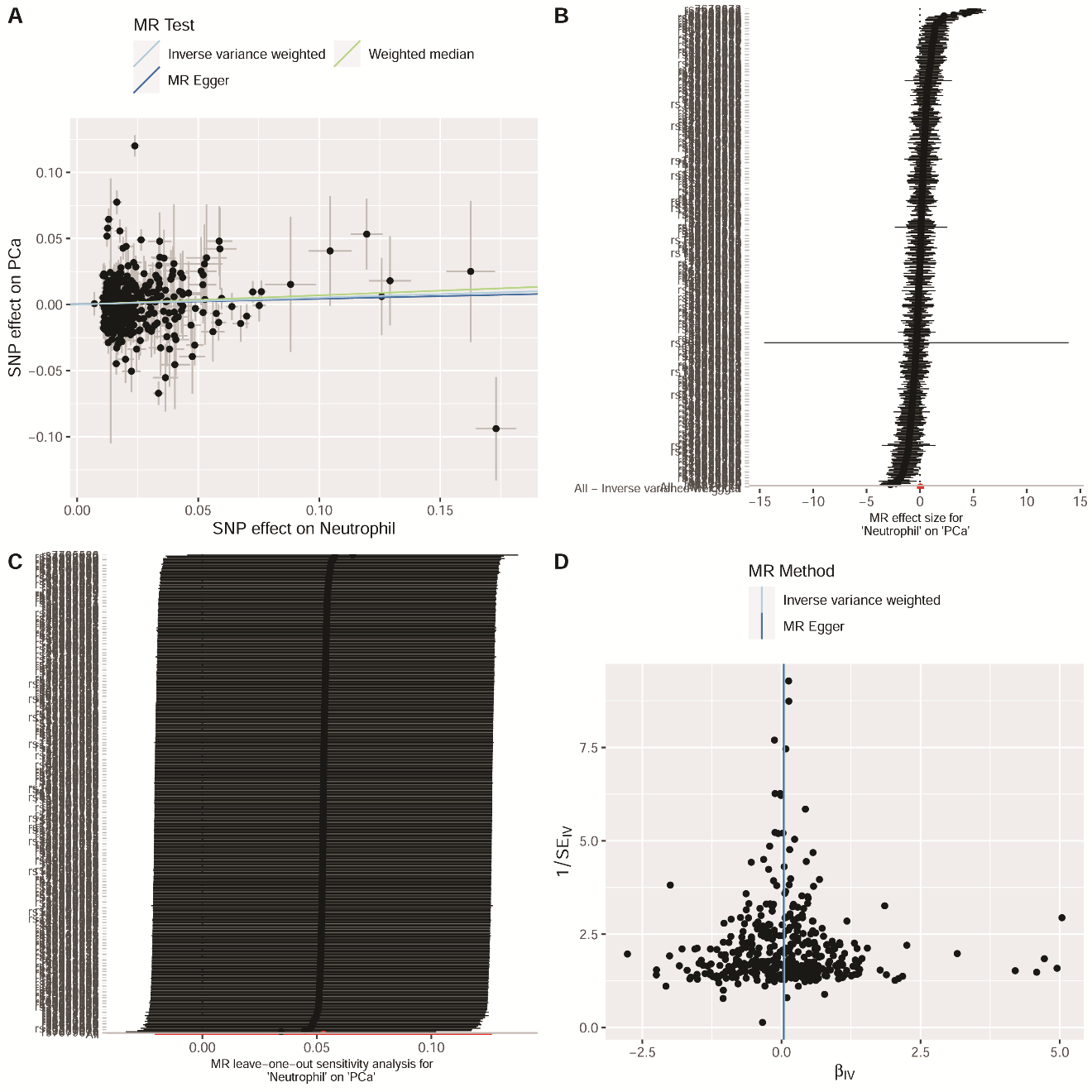


**Supplementary Figure 4.** Scatter plot (A), forest plot (B), and “leave-one-out” analysis (C) for MR analysis of Basophil Counts and PCa risk, funnel plot (D) (Primary analysis).


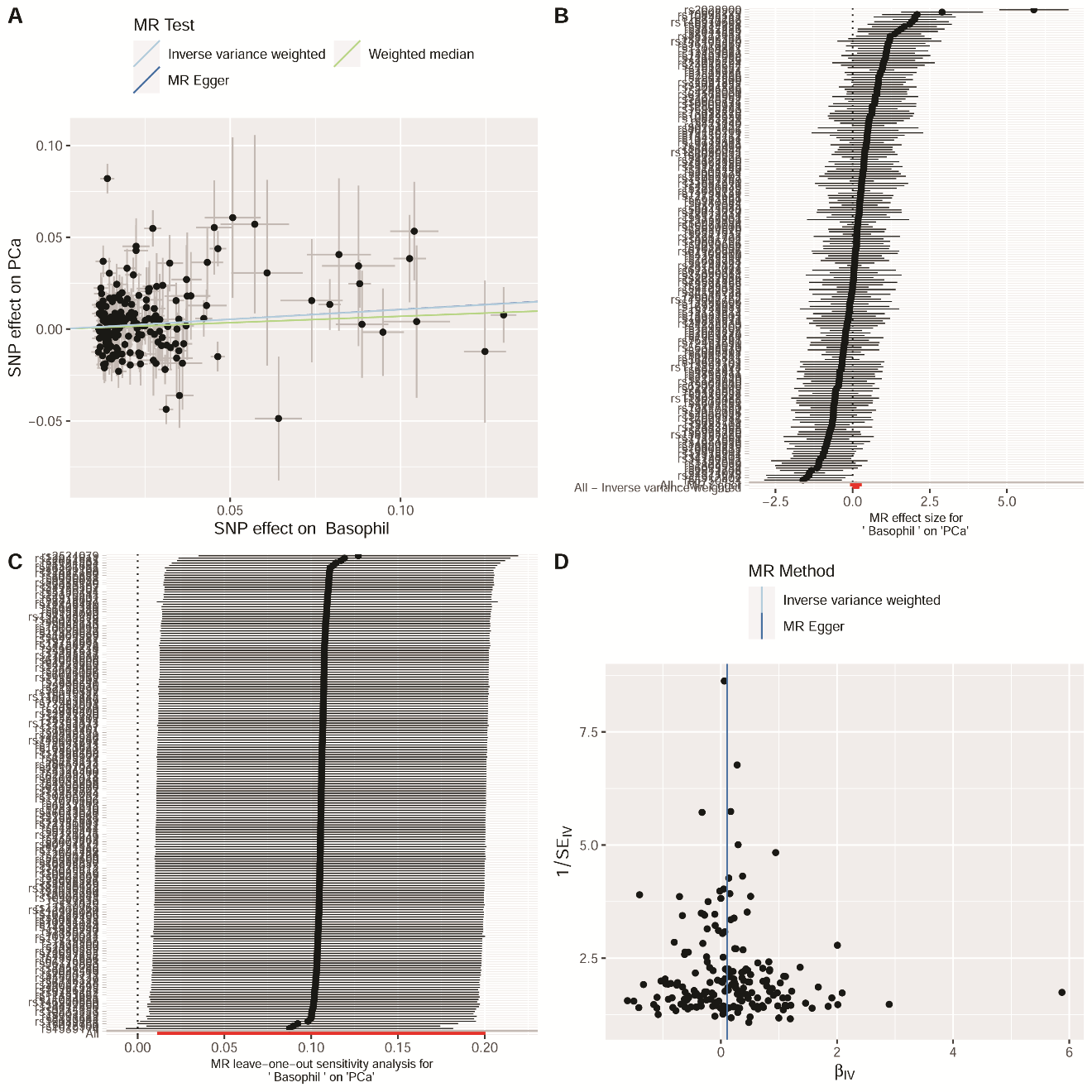


**Supplementary Figure 5.** Scatter plot (A), forest plot (B), and “leave-one-out” analysis (C) for MR analysis of Eosinophil Counts and PCa risk, funnel plot (D) (Primary analysis).


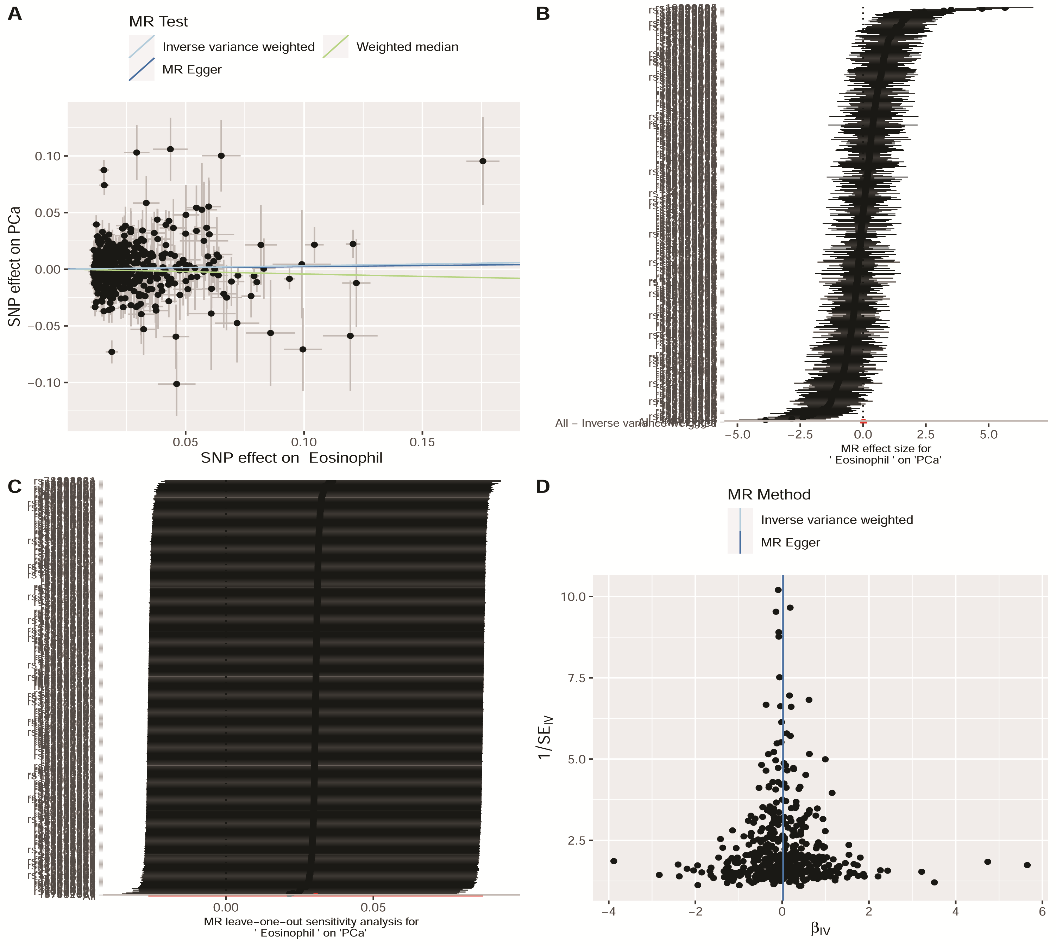


**Supplementary Figure 6.** Scatter plot (A), forest plot (B), and “leave-one-out” analysis (C) for MR analysis of Monocyte Counts and PCa risk, funnel plot (D) (Primary analysis).


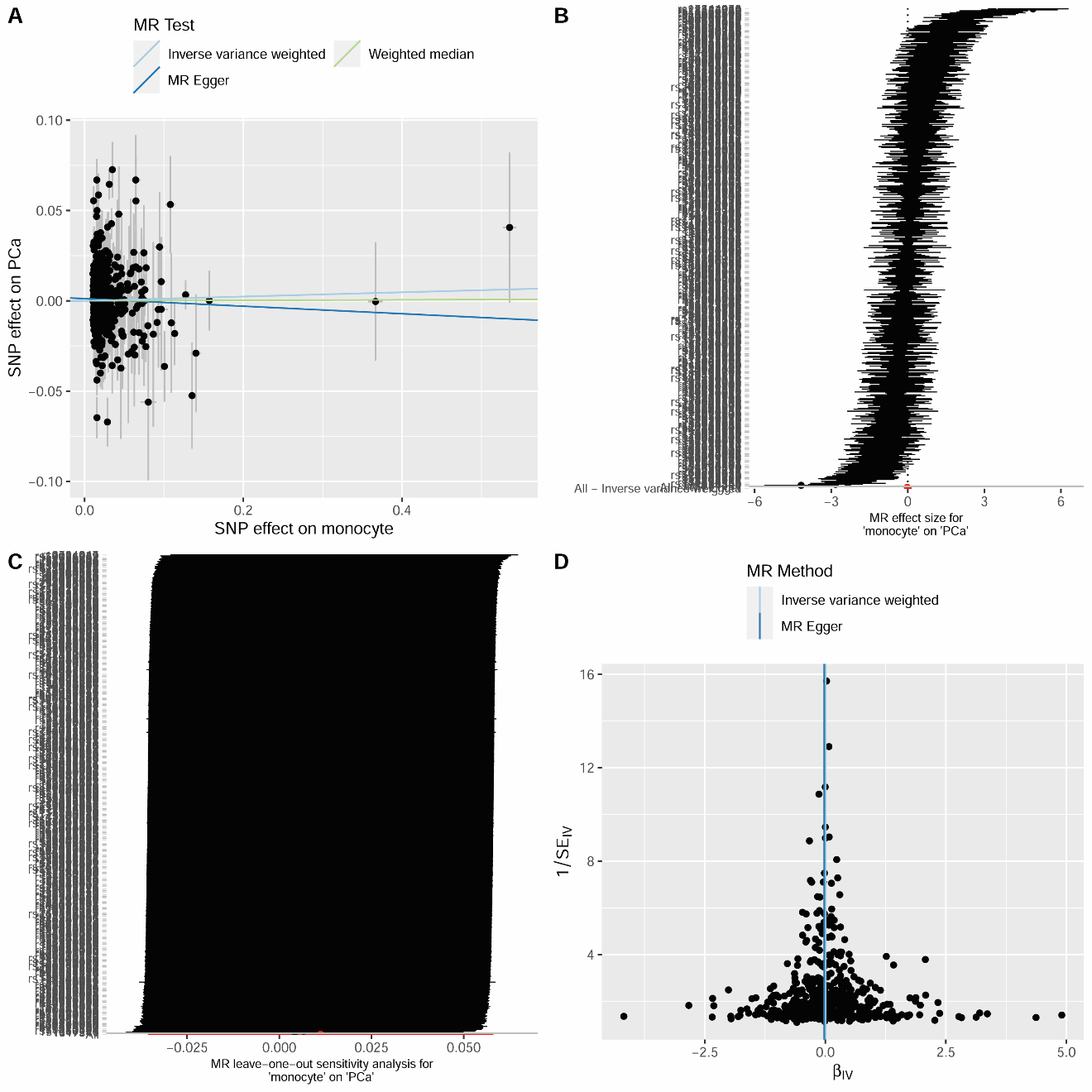


**Supplementary Figure 7.** Scatter plot (A), forest plot (B), and “leave-one-out” analysis (C) for MR analysis of Leucocyte Counts and PCa risk, funnel plot (D) (Secondary analysis).


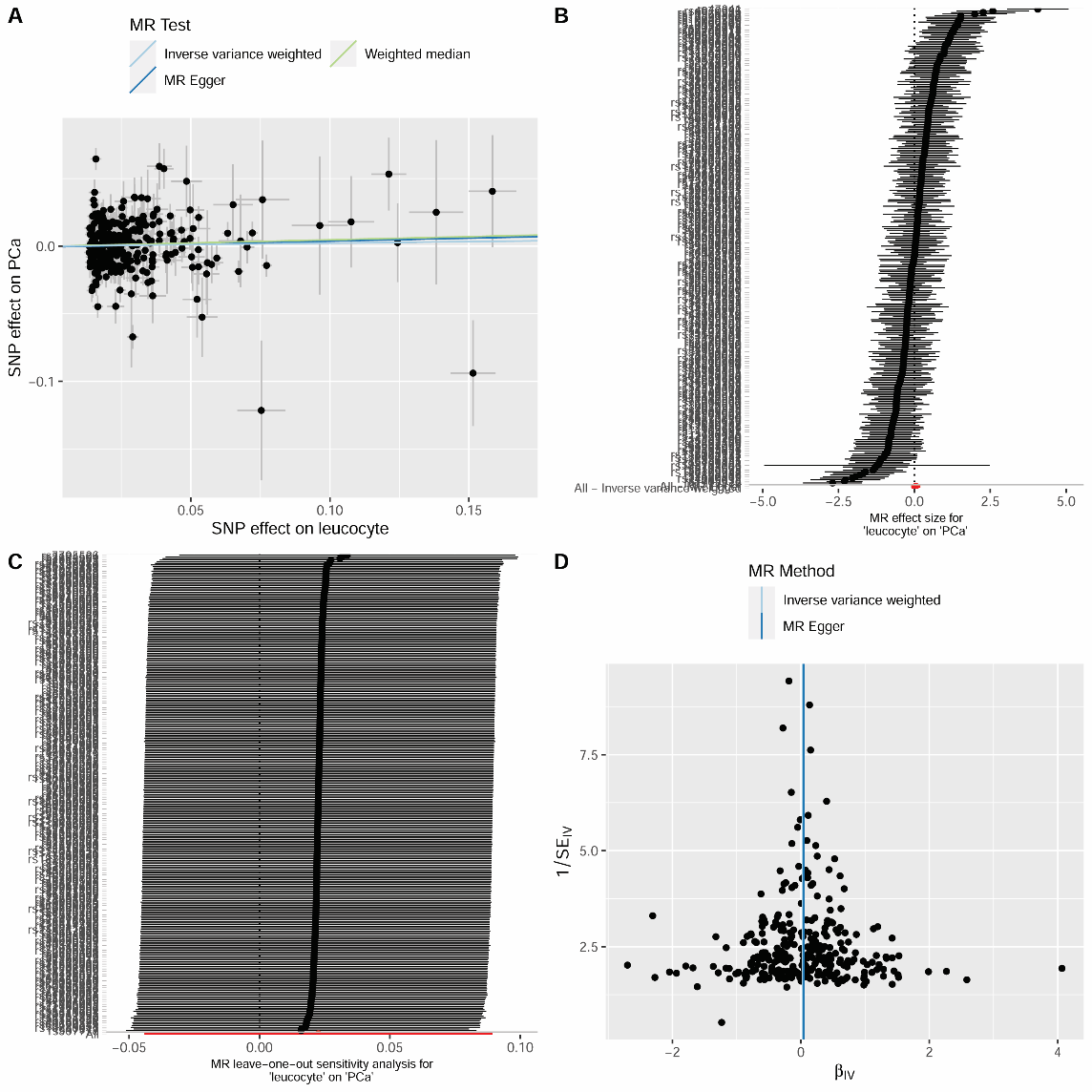


**Supplementary Figure 8.** Scatter plot (A), forest plot (B), and “leave-one-out” analysis (C) for MR analysis of Lymphocyte Counts and PCa risk, funnel plot (D) (Secondary analysis).


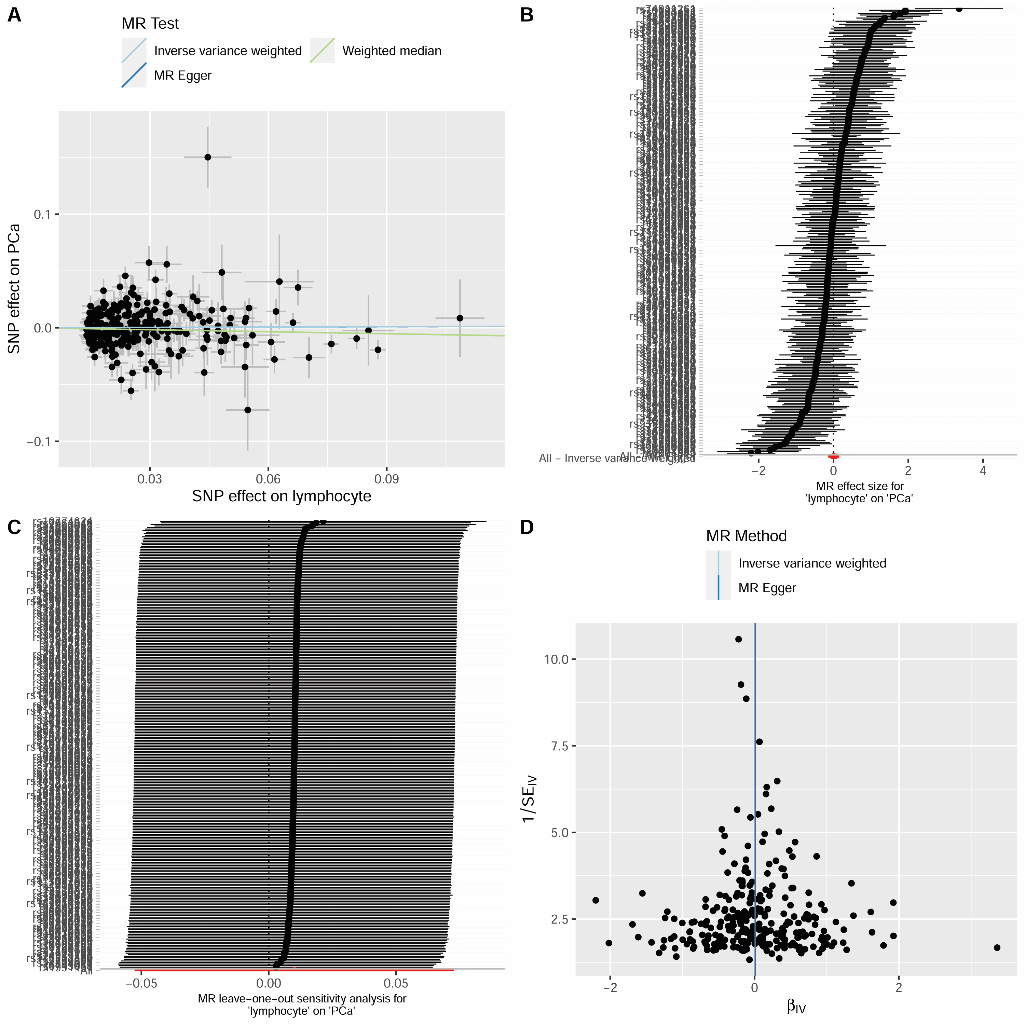


**Supplementary Figure 9.** Scatter plot (A), forest plot (B), and “leave-one-out” analysis (C) for MR analysis of Neutrophil Counts and PCa risk, funnel plot (D) (Secondary analysis).


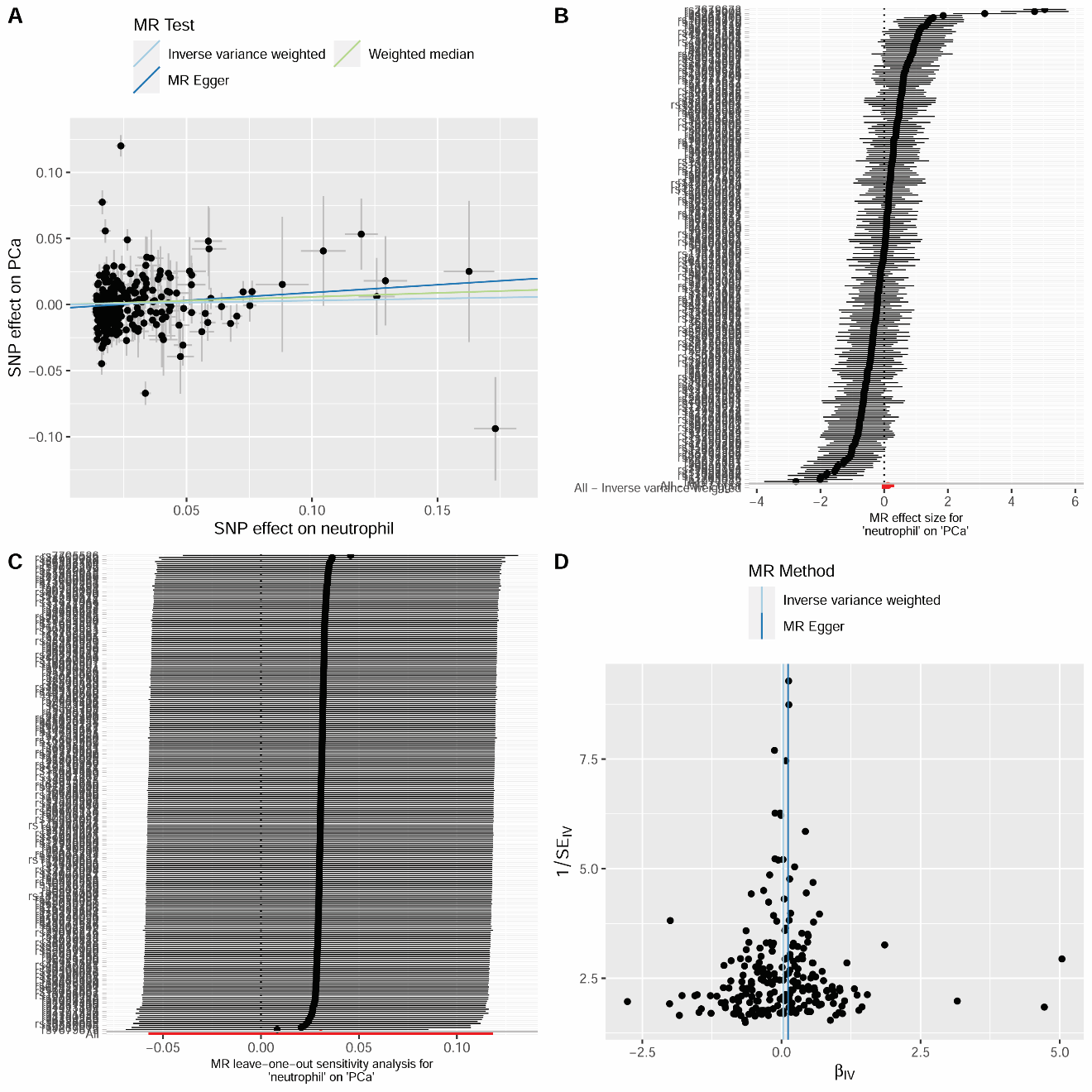


**Supplementary Figure 10.** Scatter plot (A), forest plot (B), and “leave-one-out” analysis (C) for MR analysis of Basophil Counts and PCa risk, funnel plot (D) (Secondary analysis).

***
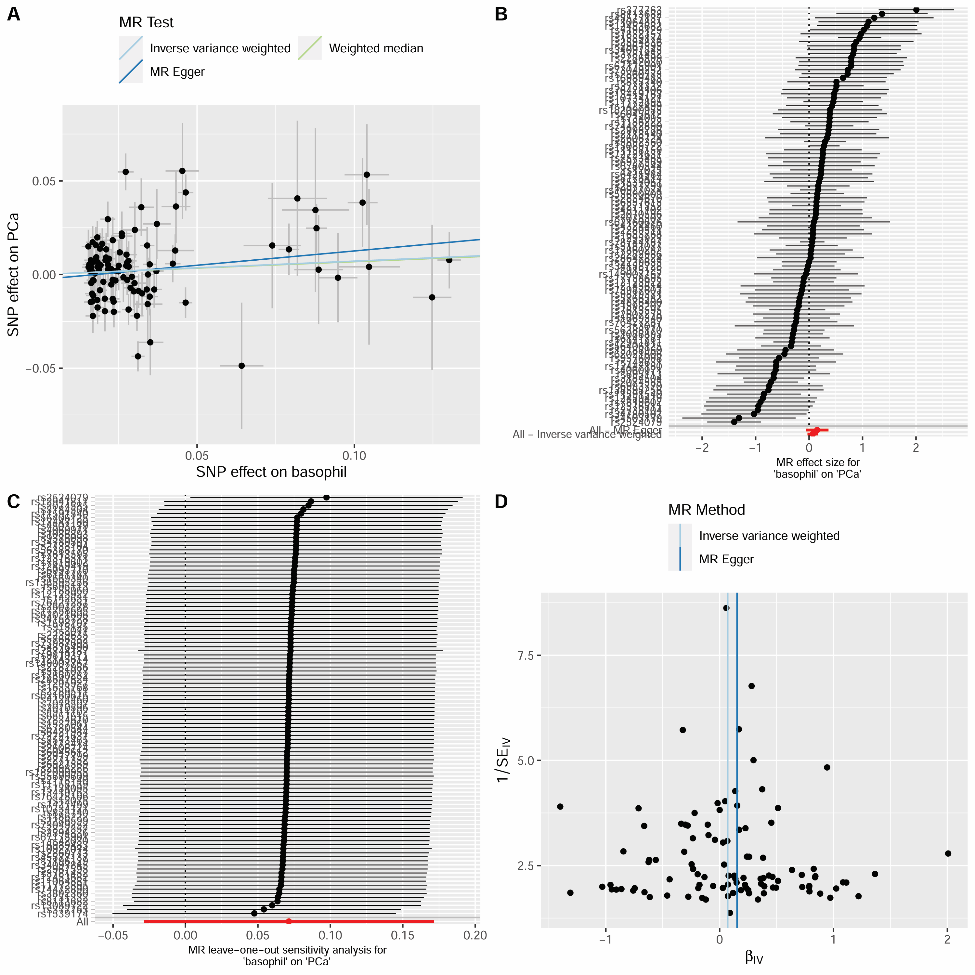
***

**Supplementary Figure 11.** Scatter plot (A), forest plot (B), and “leave-one-out” analysis (C) for MR analysis of Eosinophil Counts and PCa risk, funnel plot (D) (Secondary analysis).


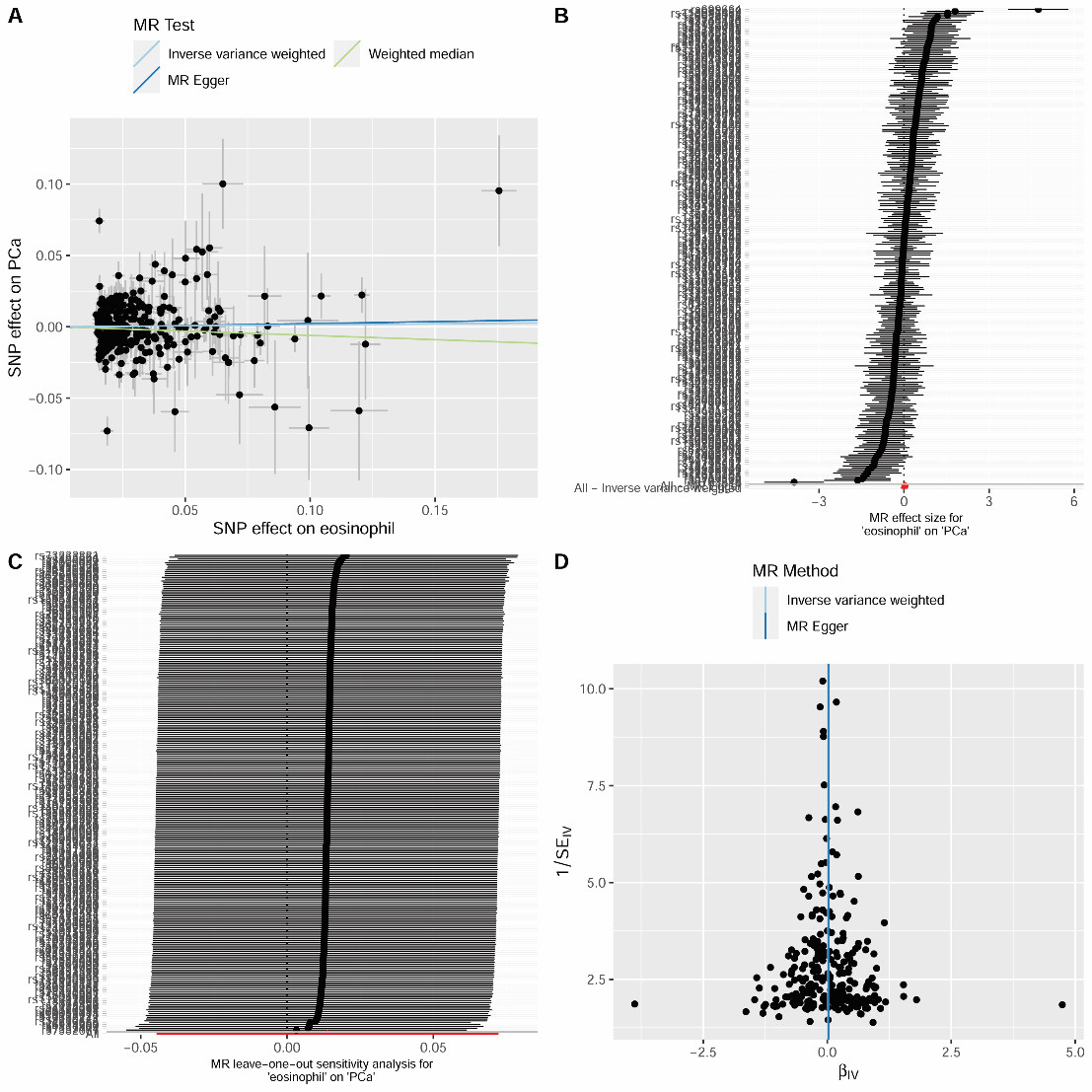


**Supplementary Figure 12.** Scatter plot (A), forest plot (B), and “leave-one-out” analysis (C) for MR analysis of Monocyte Counts and PCa risk, funnel plot (D) (Secondary analysis).


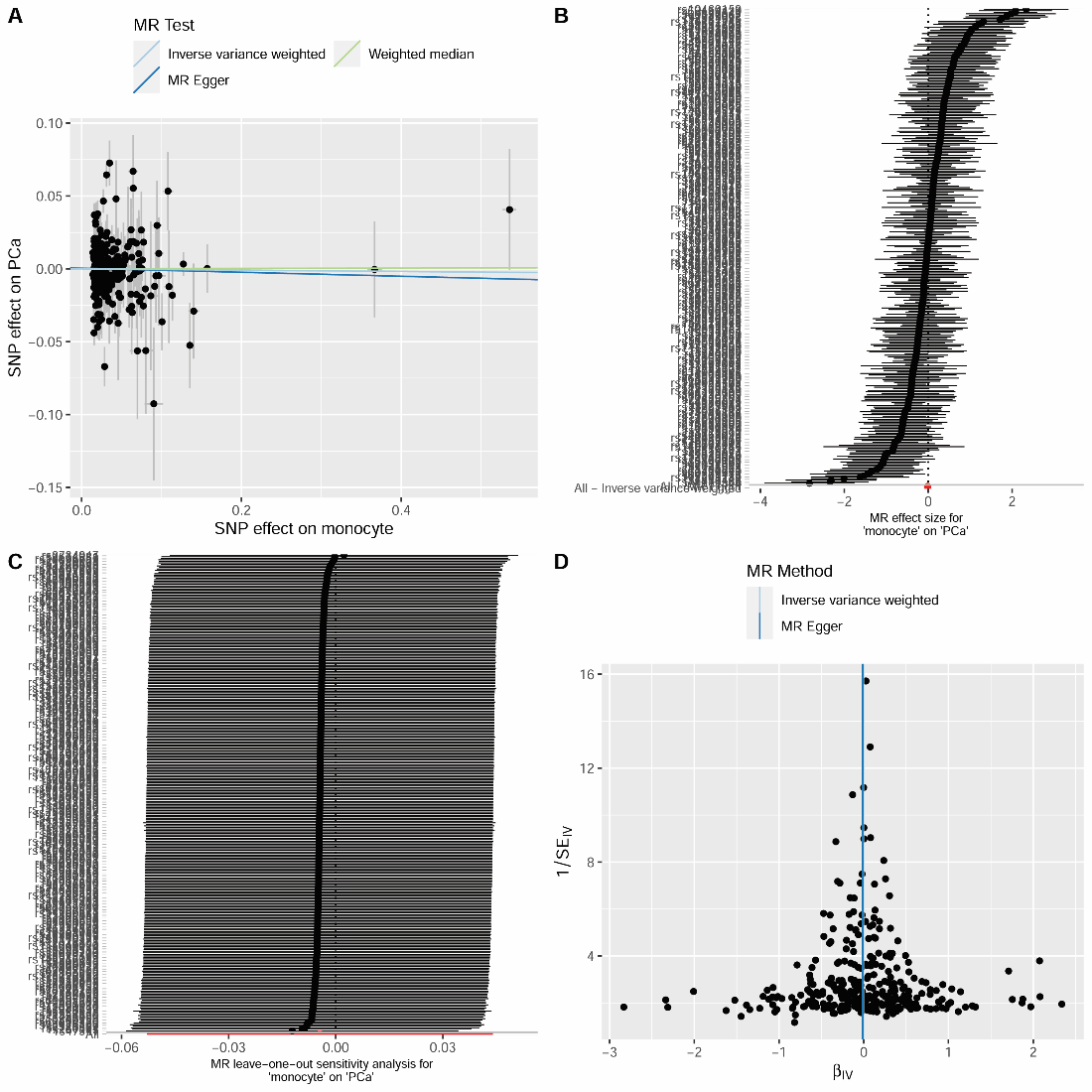


**Supplementary Figure 13.** Scatter plot (A), forest plot (B), and “leave-one-out” analysis (C) for MR analysis of PCa risk on Leucocyte Counts funnel plot (D) (Reverse analysis).


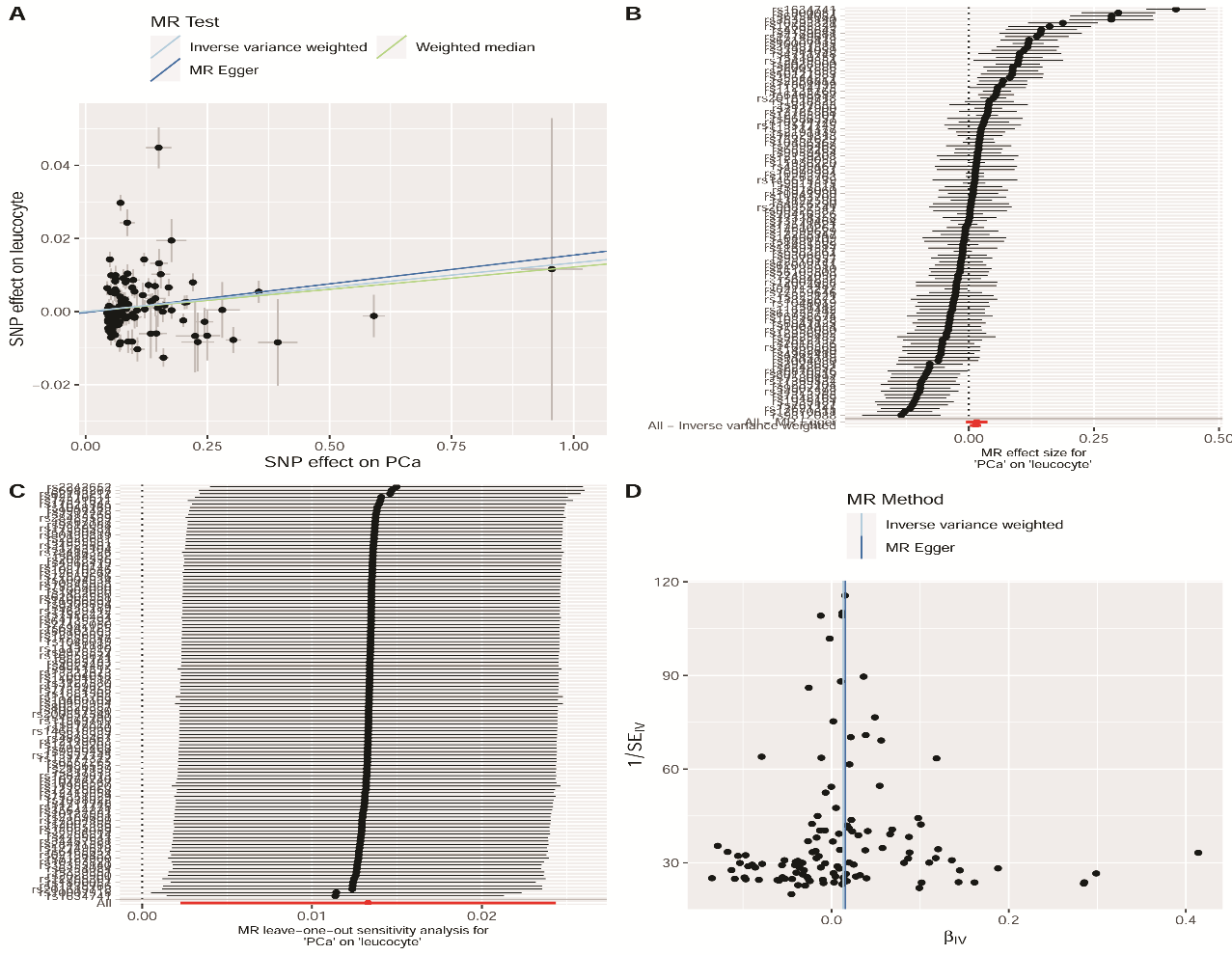


**Supplementary Figure 14.** Scatter plot (A), forest plot (B), and “leave-one-out” analysis (C) for MR analysis of PCa risk on Lymphocyte Counts, funnel plot (D) (Reverse analysis).


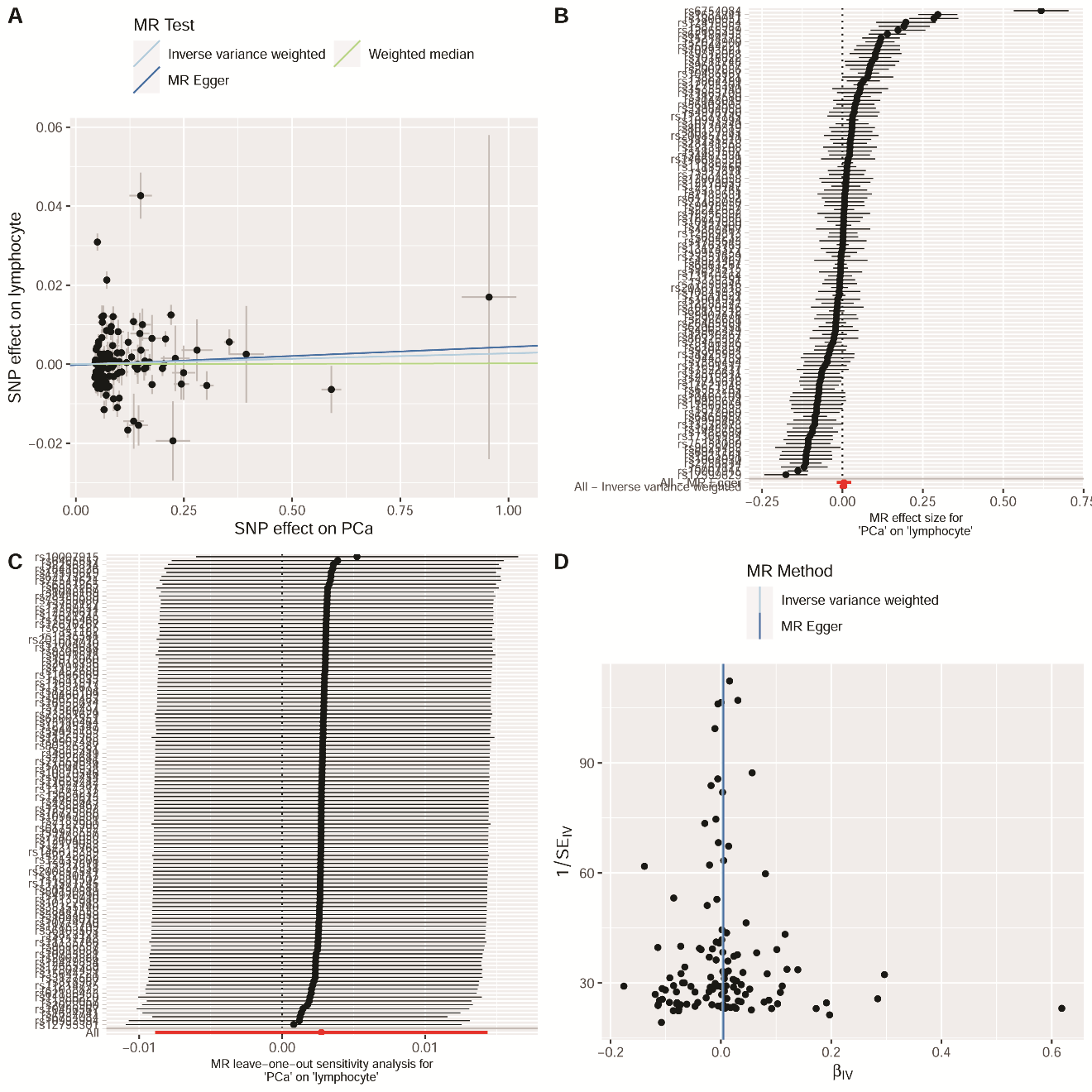


**Supplementary Figure 15.** Scatter plot (A), forest plot (B), and “leave-one-out” analysis (C) for MR analysis of PCa risk on Neutrophil Counts, funnel plot (D) (Reverse analysis).


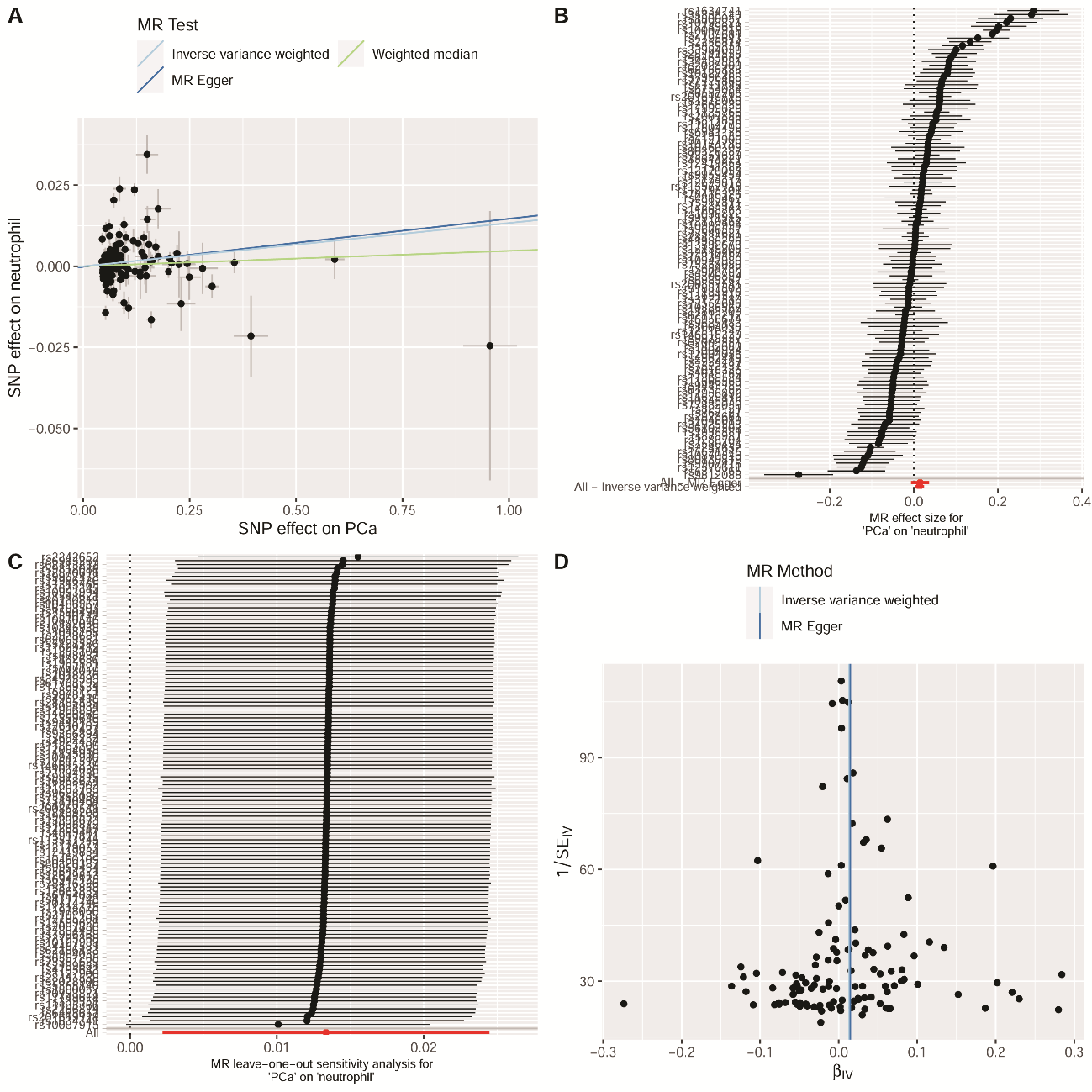


**Supplementary Figure 16.** Scatter plot (A), forest plot (B), and “leave-one-out” analysis (C) for MR analysis of PCa risk on Basophil Counts, funnel plot (D) (Reverse analysis).


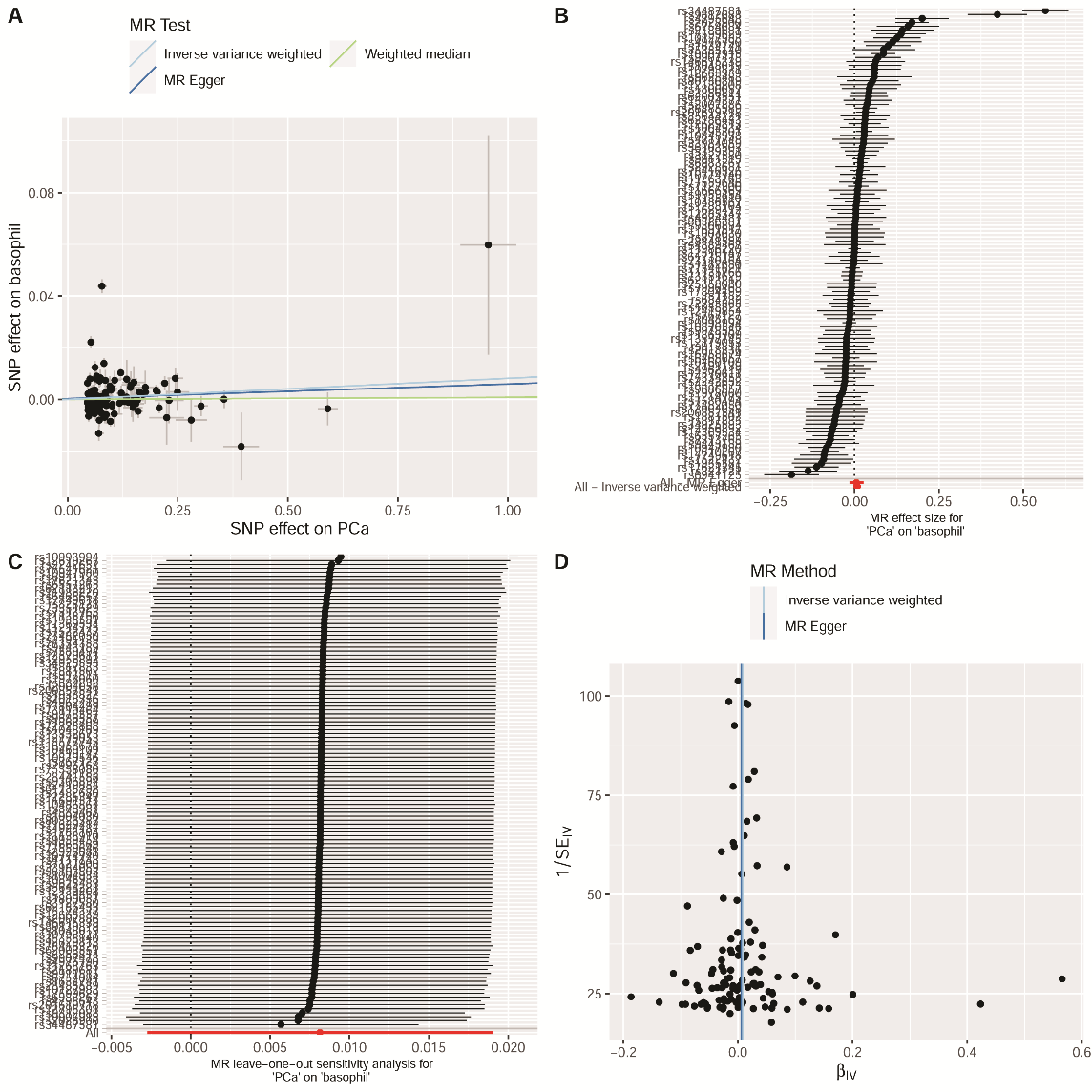


**Supplementary Figure 17.** Scatter plot (A), forest plot (B), and “leave-one-out” analysis (C) for MR analysis of PCa risk on Eosinophil Count, funnel plot (D) (Reverse analysis).


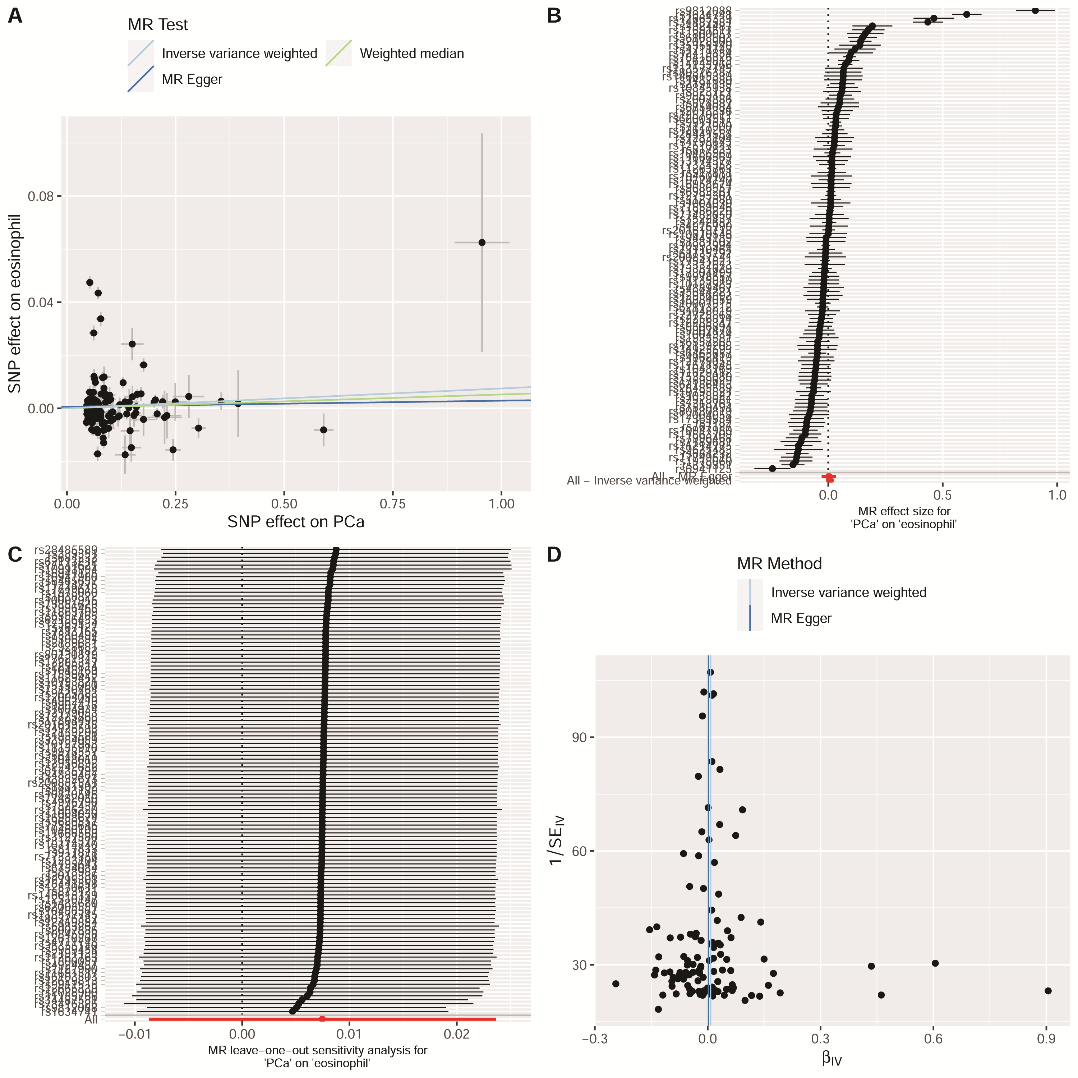


**Supplementary Figure 18.** Scatter plot (A), forest plot (B), and “leave-one-out” analysis (C) for MR analysis of PCa risk on Monocyte Counts, funnel plot (D) (Reverse analysis).


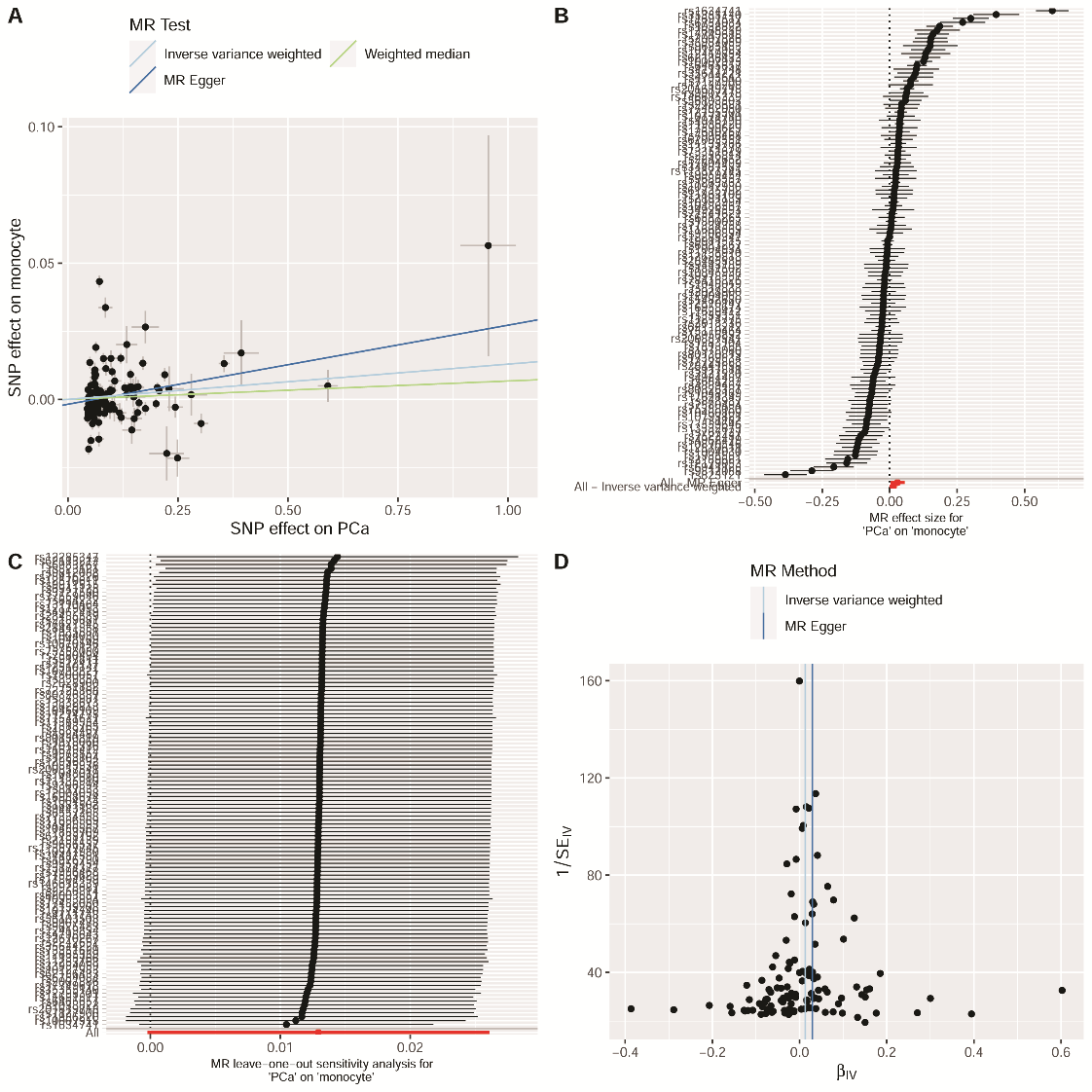

Supplement: Supplementary file 1 [file DataSheet1.docx]
